# Targeting aberrant replication and DNA repair events for treating breast cancers

**DOI:** 10.1038/s42003-022-03413-w

**Published:** 2022-05-24

**Authors:** Subapriya Rajamanickam, Jun Hyoung Park, Panneerdoss Subbarayalu, Santosh Timilsina, Kaitlyn Bates, Pooja Yadav, Saif S. R. Nirzhor, Vijay Eedunuri, Tabrez A. Mohammad, Kwang Hwa Jung, Benjamin Onyeagucha, Nourhan Abdelfattah, Raymond Benevides, Grace Lee, Yidong Chen, Ratna Vadlamudi, Andrew Brenner, Virginia Kaklamani, Ismail Jatoi, John Kuhn, Robert Hromas, Yogesh K. Gupta, Benny A. Kaipparettu, Jack L. Arbiser, Manjeet K. Rao

**Affiliations:** 1 Greehey Children’s Cancer Research Institute, The University of Texas Health Science Center at San Antonio, San Antonio, TX 78229 USA; 2 Department of Molecular and Human Genetics, Baylor College of Medicine, Houston, TX 77030 USA; 3 Department of Sciences and Mathematics, Mississippi University for Women, Mississippi, Columbus, MS 39701 USA; 4 Department of Pharmacology, The University of Texas Health Science Center at San Antonio, San Antonio, TX USA; 5 Department of Obstetrics and Gynecology, The University of Texas Health Science Center at San Antonio, San Antonio, TX 78229 USA; 6 Department of Medicine and Mays Cancer Center, The University of Texas Health Science Center at San Antonio, San Antonio, TX 78229 USA; 7 Department of Surgery, The University of Texas Health Science Center at San Antonio, San Antonio, TX 78229 USA; 8 Emory School of Medicine, Atlanta, GA 30322 USA

**Keywords:** Breast cancer, Drug development

## Abstract

The major limitations of DNA-targeting chemotherapy drugs include life-threatening toxicity, acquired resistance and occurrence of secondary cancers. Here, we report a small molecule, Carbazole Blue (CB), that binds to DNA and inhibits cancer growth and metastasis by targeting DNA-related processes that tumor cells use but not the normal cells. We show that CB inhibits the expression of pro-tumorigenic genes that promote unchecked replication and aberrant DNA repair that cancer cells get addicted to survive. In contrast to chemotherapy drugs, systemic delivery of CB suppressed breast cancer growth and metastasis with no toxicity in pre-clinical mouse models. Using PDX and ex vivo explants from estrogen receptor (ER) positive, ER mutant and TNBC patients, we further demonstrated that CB effectively blocks therapy-sensitive and therapy-resistant breast cancer growth without affecting normal breast tissue. Our data provide a strong rationale to develop CB as a viable therapeutic for treating breast cancers.

## Abstract (web summary)

A report of the small molecule Carbazole Blue demonstrates inhibition of breast cancer growth and metastasis without adverse effects on normal tissue.

## Introduction

Increasing evidence suggest that impaired replication events and alternative DNA repair pathways are major drivers in the development and progression of many cancers. Several proteins associated with the replication licensing system have oncogenic properties and act as major drivers in the development and progression of many cancers. For example, aberrant regulation of CDT1, a pre-replicative complex protein, induces continuous firing of the same origins, resulting in genomic instability and malignant transformation^1^. Furthermore, overexpression of CDC6—which is important in the assembly of the pre-replication complex at the origin of replication—is reported to promote oncogenic activities^2^. In addition, the combined expression of CDT1 and CDC6 in cells with the p53 mutation induces epithelial-mesenchymal transition and promotes invasion and metastasis^2^. Mini-chromosome maintenance genes (MCM 2,4,5; referred to as MCMs hereafter) are another example of replication licensing system proteins with oncogenic properties. MCMs unwind double-stranded DNA into a single-stranded DNA template for replication; and their activation during the cell cycle is essential for the propagation of replication^3^. Higher expression of MCM subunits has been implicated in cancer progression^4^. In addition, MCMs render cancer cells resistant to chemotherapy^5^.

Unlike normal cells, cancers rely heavily on altered DNA repair pathways for their continued proliferation. For example, cancer cells (like TNBC) with a deficiency in homologous recombination^6^ proteins (such as BRCA1) can repair their DNA by relying on other highly expressed HR-related proteins (such as RAD51 or PARP1) or on factors that support rescue DNA repair pathways such as alternative non-homologous end-joining (alt-NHEJ) that can rescue resected DNA strand breaks. These backup DNA repair pathways though ensure the uncontrolled proliferation of cancer cells but are mutation-prone, produce genetic mutations at high frequencies and contribute to therapy resistance^7^^,^^8^. Therefore, therapies aimed at targeting those rescue pathways will likely have favorable clinical outcomes. Indeed, the successful development of novel drugs such as PARP inhibitors (PARPi), which target BRCA1 and BRCA2-deficient cancers, support this proof of concept. These characteristics make repression of aberrant DNA repair pathways and impaired replication-associated proteins bona fide therapeutic strategies.

Here, we report a small molecule, Carbazole Blue (CB), that binds to DNA and blocks cancer growth and metastasis by inhibiting aberrant DNA repair events and overactive replication-associated proteins that cancer cells use to survive and progress. We derived CB from carbazole, an active ingredient of coal tar used to treat psoriasis^9^. Using ex vivo explants from estrogen receptor-positive, estrogen receptor mutant, and triple-negative breast cancer patients, we demonstrate that CB blocks breast cancer growth and metastasis without affecting normal breast tissue. Furthermore, in patient-derived xenograft (PDX) and orthotopic mouse models, systemic delivery of CB suppressed breast cancer growth and metastasis. In addition, neither short-term (4 weeks) nor long-term (2 months) treatment with CB induced toxicity in an immunocompetent preclinical mouse model. In contrast to chemotherapies that target DNA, CB appears to be a potent and safe anticancer compound. Unlike chemotherapies currently in use, which have low sequence specificity and bind indiscriminately to DNA and other macromolecules, CB may preferentially bind to a specific region/sequence of DNA and block the activity of key transcription factor/s that regulate the expression of cell cycle and DNA repair-associated genes. We show that CB blocks the activity of transcription factor HMGA1, which binds to AT-rich DNA sequences; is highly overexpressed, and supports the growth/progression of TNBC and ER + breast cancers^10, 11^. HMGA1 is reported to bind and induce the expression of gene/s that are critical for promoting cell cycle progression/DNA repair^10, 11^. Consistent with that, we show that CB inhibits the expression of several genes associated with DNA repair pathways—including RAD51 and DNA ligase I (LIG1) and consequently inhibits the heightened DNA repair activity that cancer cells employ to survive and proliferate. In addition, CB inhibited the aberrantly expressed replication-associated genes with tumor-promoting properties—including MCMs, CDC6, and CDT1. As targeting replication stress and DNA repair pathways is of growing interest, small molecules that inhibit the ability of cancer cells to repair DNA with negligible toxicity have immense potential for new breakthroughs in cancer treatments.

## Results

### Synthesis of CB

**Fig. 1:**
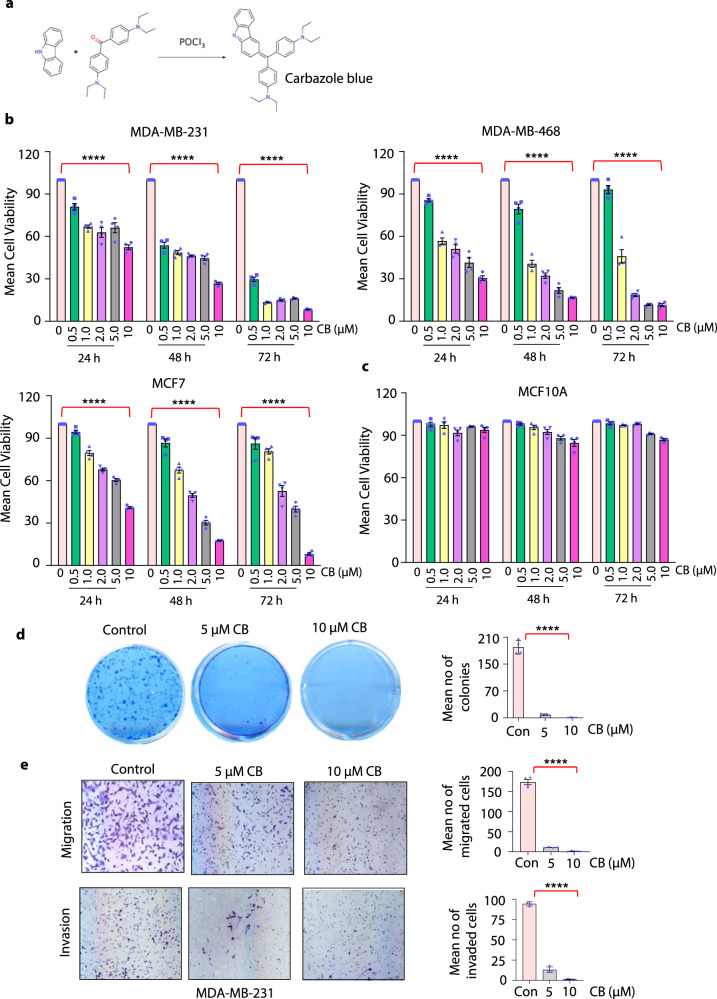
Carbazole Blue inhibits growth and migration of breast cancer cells without affecting normal human mammary epithelial cells. **a** Single-step reaction showing synthesis of Carbazole Blue (CB) using carbazole as a substrate. **b**, **c** Short-term cell viability of MDA-MB-231, MDA-MB-468, and MCF7 and normal human mammary epithelial (MCF-10A) cells (**c**) treated with vehicle control (DMSO) or indicated doses of CB (0.5–10 µM) for 24, 48, and 72 h. Cell viability was assessed using CellTiter-Glo luminescent viability assays. **d** Long-term colony formation assays of MDA-MB-231 cells were pretreated with vehicle and indicated doses of CB for 24 h and grown for an additional 7 days. Numbers of crystal violet-stained colonies were counted microscopically in ten different fields per filter. **e** Migrated and invaded MDA-MB-231 cells pretreated with vehicle or indicated doses of CB. Bar graphs show numbers of migrated and invaded cells counted microscopically in six different fields per filter. Data in **a**–**e** are mean ± SEM for at least three independent experiments. *p* values were calculated using standard Student *t*-tests. ****p < 0.0001.

We have shown that triphenylmethane pharmacophore (TPM) containing compounds contain anticancer activities^9, 12, 13^. Based on this information and by using extensive structure-activity analysis several TPM derivatives were synthesized. One such derivative is CB, which was synthesized using a single-step process in which an electrophilic addition occurs at the ortho or para position to hydroxy or amine groups in the phenol or aniline compounds (Fig. 1a). The structure and absorption spectra of CB were confirmed by nuclear magnetic resonance spectroscopy and absorbance spectrophotometry, respectively (Supplementary Fig. 1a, b). The molecular weight of CB was determined to be 588 g/mol. Our initial in vitro screen with CB, its parent compound carbazole as well as other derivatives showed that CB had the most potent anti-growth effect on breast cancer cells (Supplementary Fig. 1c).

### CB inhibits the viability and invasive capabilities of breast cancer cells without affecting normal mammary epithelial cells

To do a more thorough analysis of the effect of CB on breast cancer growth, we performed short and long-term viability in several breast cancer cell lines (MDA-MB-231, MDA-MB-468, BT-549, ZR75-1, SKBR3, and MCF7). Breast cancer cells treated with varying concentrations of CB showed time- and dose-dependent decreases in cell proliferation (Fig. 1b; Supplementary Fig. 2a; and Supplementary Data 1). However, CB did not affect the proliferation of normal mammary epithelial cells (HMEC and MCF-10A) (Fig. 1c and Supplementary Fig. 2b). In addition, CB treatment inhibited the long-term viability of breast cancer cells (Fig. 1d and Supplementary Fig. 2c). Next, we asked whether CB may also inhibit migration and invasion of breast cancer cells. Trans-well assays on breast cancer cells treated with CB for 6 h (a time point which does not have a significant effect on cell viability) showed that CB inhibited invasion and migration of all breast cancer cells in a dose-dependent manner (Fig. 1e and Supplementary Fig. 3a, b). In addition to breast cancers, we found that CB inhibited the growth of prostate, lung, and endometrial cancer cells suggesting that CB may have antitumor effects in multiple cancers (Supplementary Fig. 4).

### Liposomal nanocarrier encapsulation of CB and in vivo pharmacokinetics

**Fig. 2:**
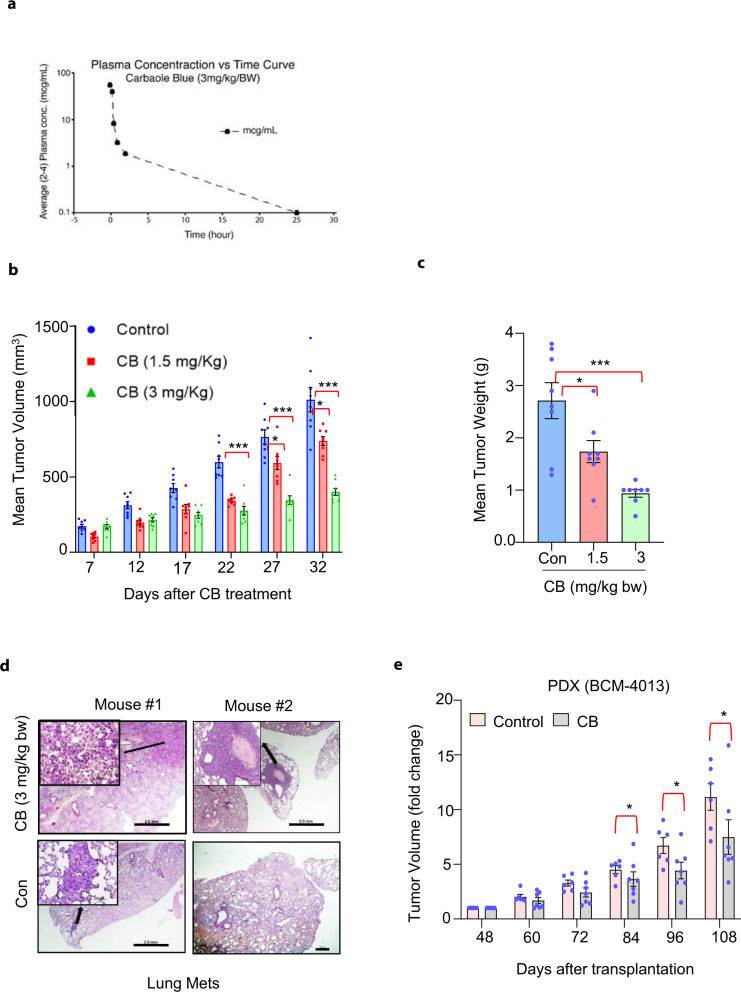
Therapeutic potential of CB. **a** Plasma concentrations of CB for indicated time periods after single intraperitoneal injection of CB (3 mg/kg b.w.) conjugated with 20% intralipid (molar drug/lipid ratio of 0.07) in mice (*n* = 2–4/group). **b** Mean tumor volume in vehicle- or CB conjugated with nanoparticle-treated mice. MDA-MB-231 cells were subcutaneously implanted into mammary fat pads of athymic nude mice. After tumors reached ~100 mm^3^, mice were treated with either vehicle or CB (1.5 and 3 mg/kg body weight) conjugated with intralipid, every 5 days for 30 days. **c** Tumor weight in vehicle- and CB-intralipid treated mice (mean ± SEM; *n* = 8/group). **d** Representative lung sections from two representative mouse (stained with hematoxylin and eosin [H&E] from a vehicle and CB-intralipid treated mice. Arrows in the inset show metastatic lesions. Scale bar, (2X, 2.0 mm and insert-20 × 50 μm). **e** Mean tumor volume in xenografts derived from a patient with stage III triple-negative breast cancer patient (BCM-4013) treated with vehicle or CB. Tumors were transplanted to the fourth mammary glands of mice (*n* = 6/group). After the tumors reached around 100 mm^3^, mice were treated with vehicle and CB conjugated with nanoparticles (3 mg/kg b.w.) starting from day 48 after the tumor transplantation. Data in **b**, **c**, **e** are mean ± SEM. *p* values were calculated using standard Student *t*-tests. *p < 0.05; ***p < 0.0001.

Before testing the therapeutic efficacy of CB in vivo, we determined the circulation half-life of CB and its potential be delivered systemically. For systemic delivery, we encapsulated CB with intralipid (an FDA-approved emulsion for delivery), since our previous studies suggested that doing so increases the circulation half-life of anticancer agents^14, 15^. To confirm that this is also true for CB, we directly loaded CB (3 mg/kg bw) in 20% intralipid (nano-CB) with a molar drug/lipid ratio of 0.07 and administered it intraperitoneally to adult female BALB/c mice (5–6 weeks old). The plasma concentration versus time curve revealed a peak CB concentration of 57.48 μg/ml immediately post-injection. The estimated elimination constant (kel) was 0.129 h, resulting in a calculated (0.693/kel) half-life of 5.37 h (Fig. 2a). Next, we tested whether nano-CB retained the anticancer effects of CB. Nano-CB significantly inhibited the viability of breast cancer cells compared to control (Supplementary Fig. 5).

### Therapeutic potential of CB

To test CB’s anticancer activity in vivo, we used orthotopic xenograft and PDX models. For the orthotopic model, MDA-MB-231 cells were implanted into the mammary fat pad of athymic nude/SCID mice followed by treatment with specified concentrations of CB or vehicle control after a week, when tumors reached approximately 150 mm^3^ size. Nano-CB (1.5 and 3 mg/kg bw) was injected (intravenously) once a week for 4 weeks. Nano-CB caused marked inhibition of mammary tumor growth in a dose-dependent manner compared to vehicle control (Fig. 2b, c). To determine if CB may also inhibit metastasis, we monitored a group of tumor-bearing athymic nude/SCID mice for additional 3 weeks. Vehicle-treated mice showed aggressive multifocal metastasis, while CB-treated mice showed either no lung lesions or much smaller metastatic foci (Fig. 2d).

To further validate our in vivo results, we tested CB’s potential to inhibit tumor growth in a PDX (BCM-4013) model. PDX generated from triple-negative breast cancer patients were implanted into mammary fat pads of old SCID/Beige female mice When the tumors reached ~100 mm^3^, the mice were treated with nano-CB every 4th day for 9 weeks. The CB treatment significantly reduced the tumor growth of the PDX model (Fig. 2e).

### CB is a safe and viable anticancer compound

**Fig. 3:**
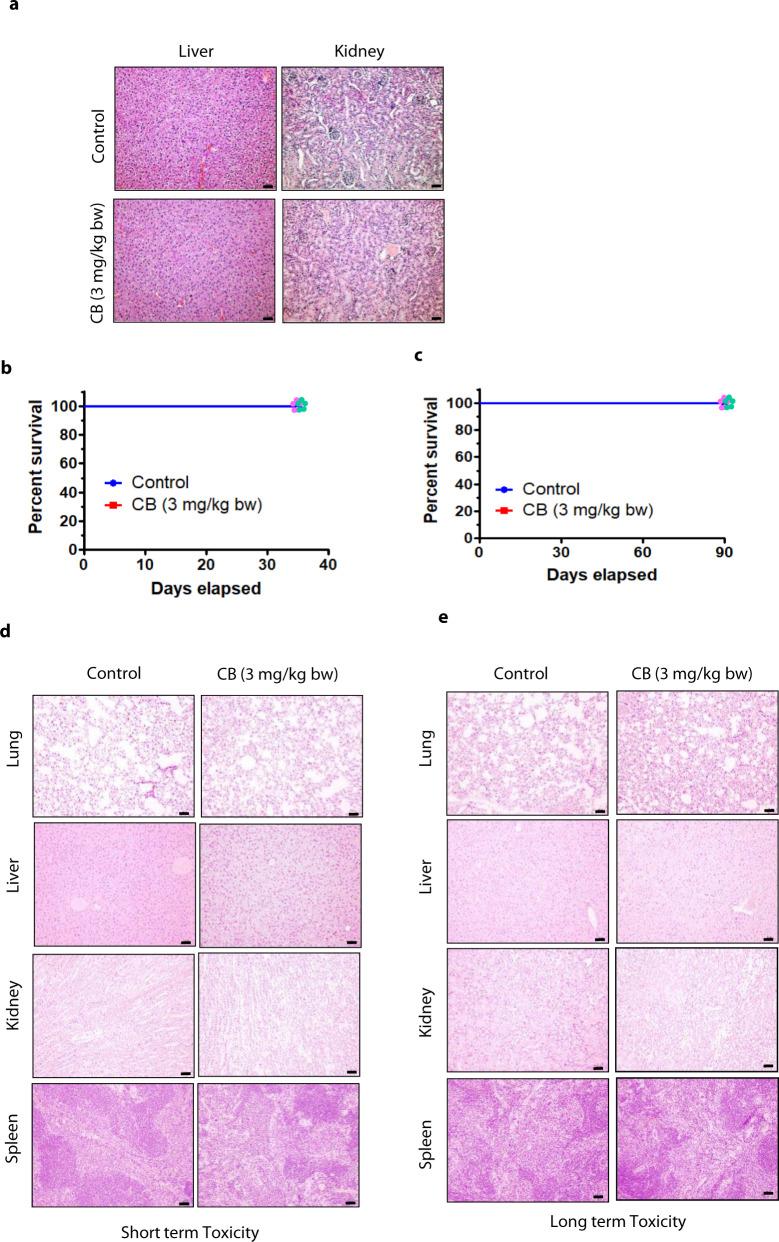
CB is a safe and viable anticancer compound. **a** Representative H&E-stained sections of livers and kidneys from CB-intraipid (nano-CB)-treated mice show no signs of liver and renal toxicity in an orthotopic xenograft model. Scale bar, (10X, 10 µm). **b**, **c** Survival of 4–5 weeks old BALB/cJ mice treated with vehicle (Control) or CB-intralipid for short or long durations. Mice received vehicle or nano-CB (3 mg/kg b.w., intravenously; *n* = 5/group) once per week for 4 weeks. **b** One set of mice was sacrificed after 24 h of final CB administration. **c** For long-term toxicity studies, mice were followed for an additional 2 months. **d**, **e** Representative H&E-stained sections of lungs, livers, kidneys, and spleens from BALB/cJ mice treated with vehicle ^24^ or nano-CB for short- (**d**) and long-term (**e**). Scale bar, (10X, 10 µm).

To determine whether CB is a safe anticancer compound, we tested vehicle- and CB-treated mice in both orthotopic and PDX tumor models. No overt signs of toxicity were observed in CB-treated mice; body weights did not change, and cell morphology from different organs showed no changes compared to vehicle-treated tumor-bearing mice (Fig. 3a and Supplementary Fig. 6a, b). To further confirm these results, we performed short and long-term toxicity studies in immunocompetent BALB/cJ mice. For both short- and long-term toxicity studies, BALB/cJ mice received nano-CB (3 mg/kg b.w. intravenously) once a week for 4 weeks. To evaluate short-term toxicity, one set of mice was sacrificed within 24 h of the final CB dose. For long-term toxicity studies, mice were followed for an additional 2 months. CB-treated Balb/C mice showed no difference in survival compared to the vehicle-treated group (Fig. 3b, c). In addition, histologic analysis of tissues from CB-treated mice showed no changes in cellular morphology from any organ, including lung, liver, kidney, and spleen, suggesting no toxicity (Fig. 3d, e and Supplementary Fig. 7). Furthermore, CB had no effect on the hematopoietic system as revealed by no change in the levels of CD3, CD4, CD8, and CD44 in CB-treated mice compared to vehicle-treated mice (Table 1). These results suggest that CB could be a safe and potent treatment option for both primary and metastatic breast cancers.

**Table 1:** FACS analysis showing numbers in immune cells isolated from.

	Control	CB
**CD3**+	27.58 ± 1.69	29.02 ± 1.32
**CD4**+	73. 5 ± 0.74	72.88 ± 5.56
**CD8**+	17.84 ± 0.81	15.1 ± 1.98
**CD44**+	98.32 ± 0.23	81.8 ± 17.10

Spleen of Balb/c mice treated with CB (3 mg/kg bw; *n* = 5) or vehicle (*n* = 5) for 40days).

*BW* body weight.

### CB is safe and effective against human breast cancers

**Fig. 4:**
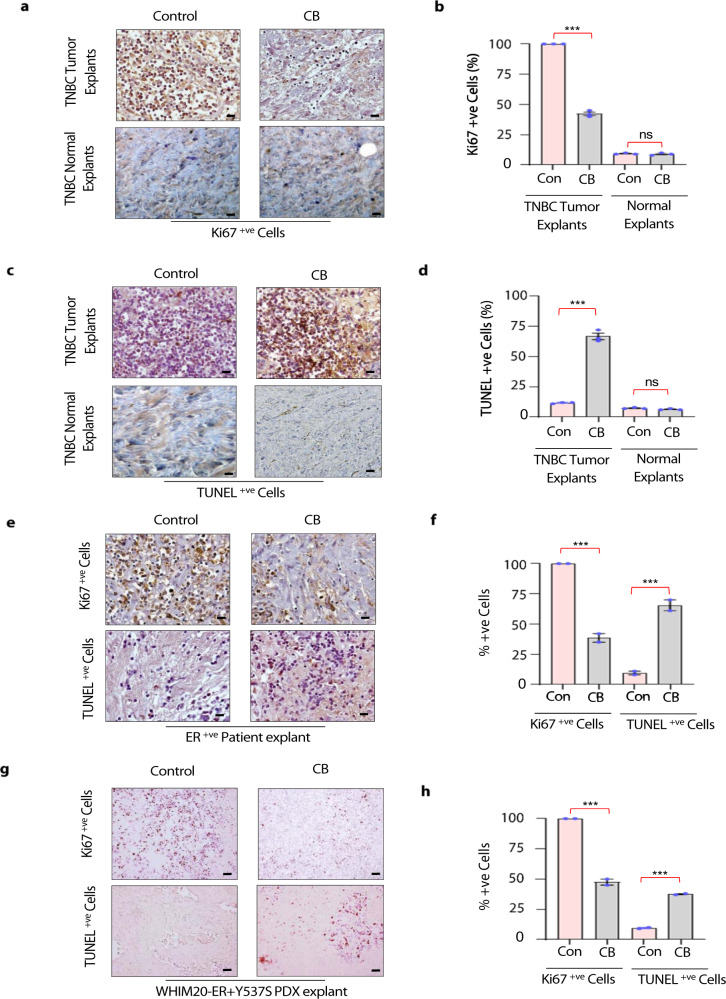
CB is a safe and effective therapeutic regimen for treating human breast cancers. **a** Representative images showing immunohistochemical analysis using Ki67 antibody in triple-negative breast cancer (TNBC) patient-derived tumor explants and normal adjacent tissues treated with either nano (intralipid)-vehicle or nano (intralipid)-CB for 72 h. Representative photographs from one TNBC patient are presented at 40x (Total *n* = 3 TNBC patients). Scale bar, (10X, 10 µm). **b** Average number of Ki67-positive cells derived from six randomly selected microscopic fields from different explants derived from TNBC patients. Ki67 score was defined as the percentage of positively stained cells among the total number of malignant cells scored. Scoring was done in whole tumor sections. **c** Representative images showing TUNEL assay on TNBC patient-derived cancer and normal adjacent tissues treated with either nano-vehicle or nano-CB for 72 h. Scale bar, (10X, 10 µm). **d** Average numbers of TUNEL-positive cells derived from six randomly selected microscopic fields from different explants derived from TNBC patients as described in **b**. **e** Representative images showing Ki67 staining and TUNEL assay on explants derived from two ER + breast cancer patients and treated with either nano-vehicle or nano-CB for 72 h. **f** Average number of Ki67 and TUNEL -positive cells derived from six randomly selected microscopic fields from each of two different explants. Ki67 and TUNEL- positive cells were scored as described in **b** and **d**. **g** Representative images showing Ki67 staining and TUNEL assay on ER mutant WHIM20-ER + Y537S PDX explants treated with either nano-vehicle or nano-CB for 72 h. **h** Average number of Ki67 and TUNEL -positive cells derived from six randomly selected microscopic fields from each of two different explants. Ki67 and TUNEL- positive cells were scored as described in **b**, **d**. Scale bar in a, c, e, and g: 10X, 10 µm. Data in **b**, **d**, **f**, and **h** are mean ± SEM. *p* values were calculated using standard Student *t*-tests ***p < 0.001.

We then tested whether CB would indeed be a safe and viable therapeutic alternative for breast cancer patients. To address this question, we tested the efficacy of CB in ex vivo explants using tumor tissues collected from triple-negative breast cancer and estrogen receptor (ER) positive breast cancer patients. Recently, we demonstrated that ex vivo explants recapitulate the structural complexity and individual heterogeneity of human breast cancers^15^ and therefore can enable the evaluation of drug efficacy in a tumor’s native 3D microenvironment (Supplementary Fig. 8a). TNBC and ER-positive tumor explants were treated with vehicle or nano-CB for 72 h and subjected to immunohistochemical analysis using an antibody against Ki67 and TUNEL apoptosis assays. Nano-CB inhibited cell proliferation and induced apoptosis in tumor tissues from patient explants, as revealed by significantly reduced Ki67 levels (Fig. 4a, b) and significantly increased TUNEL staining in CB-treated explants (Fig. 4c, d). Importantly, CB did not affect the growth of normal cells, as revealed by comparable Ki67 levels and the absence of apoptotic cells in untreated and nano-CB-treated explants from normal adjacent mammary tissues (Fig. 4a–d). We observed similar trends in ER + breast cancer patient explants (Fig. 4e, f and Supplementary Fig. 8b). Next, we tested whether CB may also be effective against ER mutant breast cancers that show therapy resistance. To study that, we tested the effect of CB on tumor explants from ER mutant (WHIM20-ER + Y537S) PDXs. Substitution of tyrosine at position 537 to serine (Y537S) in the ligand-binding domain of estrogen receptor 1 (ESR1*)* is reported to constitutively activate ER in a ligand-independent manner^16, 17^. In particular, Y537S ESR1 mutation is an important driver of endocrine-refractory ER + metastatic breast cancers^16^. Our results demonstrated that CB treatment inhibited proliferation and induced apoptosis in WHIM20-ER + Y537S PDX explants (Fig. 4g, h). Collectively, our findings indicate that CB may be a safe therapeutic regimen for treating therapy-sensitive and therapy-resistant breast cancers.

### CB targets genes associated with DNA replication, cell cycle progression, and DNA damage surveillance pathway

**Fig. 5:**
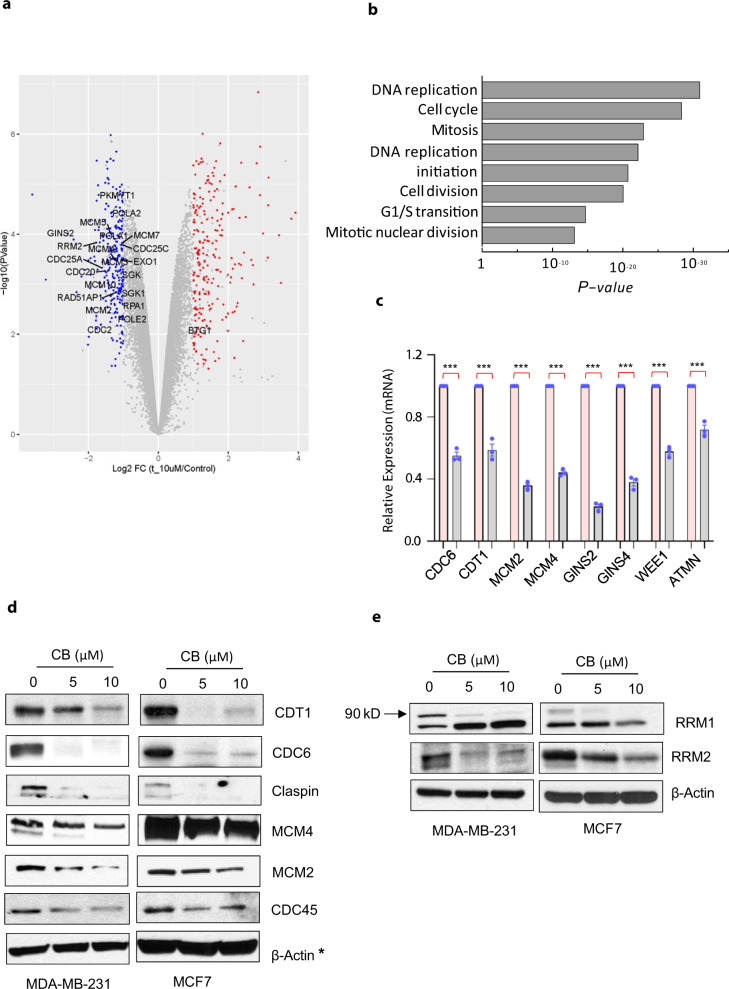
CB regulates the expression of genes associated with DNA replication, cell cycle progression, and DNA damage surveillance pathway. **a** Gene expression changes in vehicle- (Control) or CB-treated MDA-MB-231 cells. Cutoff criteria for differentially expressed genes included absolute log2 fold change >1, and *p* value < 0.05 (red color indicates an increase and blue color represents a decrease in mRNA expression). **b** Results of Ingenuity pathway analysis showing top-ranked biological pathways altered in CB-treated MDA-MB-231 cells. **c** Real-time qPCR validation of highly altered genes in vehicle and CB-treated MDA-MB-231 breast cancer cells using gene-specific primers. Relative expression of each gene was quantified by measuring Ct values and normalized with GAPDH. Results are shown as mean ± SEM for at least three independent experiments. *p* values were calculated using standard Student *t*-tests. ***P < 0.001. **d**, **e** Western blots of MDA-MB231 and MCF7 cells treated with either vehicle or CB for 24 h using antibodies against indicated proteins. Membranes were reprobed with different antibodies and with β-actin, which served as a loading control. The blots shown are representative of at least three independent experiments. * symbols next to β-actin indicate the same loading control as in Fig. 7g.

To elucidate how CB may inhibit cancer growth and progression, we performed gene expression analyses on breast cancer cells treated with or without CB (Fig. 5a). Ingenuity Pathway Analysis (IPA) of differentially expressed genes revealed that genes involved in the replication, cell cycle, and DNA repair pathways were highly enriched (Fig. 5b). Examples of those genes include chromatin licensing and DNA replication factor 1 (*CDT1*), cell division cycle 6 (*CDC6*), mini-chromosome maintenance genes (*MCM2,3,4,5,6,7*), cell division cycle 45 (*CDC45*), ribonucleotide reductase catalytic subunit M1 and M2 (*RRM1/RRM2*), DNA polymerase epsilon (*POLE2,3*), GINS complex unit 2/3 (*GINS2, GINS3*), *RAD51* and *LIG1* (Fig. 5a). These target genes were significantly downregulated, both at the RNA and protein levels, in CB-treated MDA-MB-231, MDA-MB-468, and MCF7 cells (Fig. 5c, d, e and Supplementary Figs. 9a, b, c,  13–22 (unprocessed blots)). To further substantiate these findings, we determined the levels of target genes in ex vivo explants from breast cancer patients. The expression of CB target genes were significantly reduced in patient-derived explants compared with vehicle-treated tumors (Supplementary Fig. 9d). Importantly, a meta-analysis of the TCGA data set showed that CB target genes are highly overexpressed in tumors of breast cancer patients compared to normal adjacent control tissue (Supplementary Fig. 10). These results strongly suggest that inhibition of replication and DNA repair-associated genes may be one of the mechanisms by which CB imparts its antitumor activities.

**Fig. 6:**
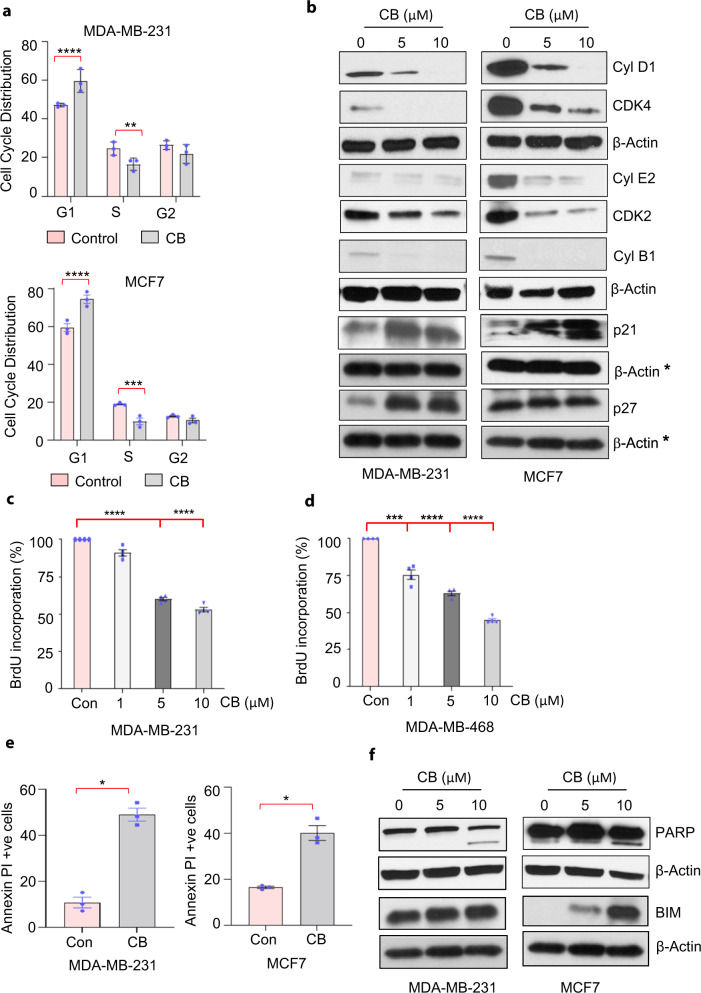
CB blocks cell cycle progression and induces apoptosis in breast cancer cells. **a** Cell cycle distribution of vehicle and CB-treated MDA-MB-231 and MCF7 cells. Breast cancer cells were treated with vehicle or CB for 48 h, stained with propidium iodide, and analyzed for cell cycle distribution by flow cytometry. Data shown are mean ± SEM of three samples for each treatment and represent three independent experiments. **b** Western blots of MDA-MB-231 and MCF7 cells treated with vehicle or CB using antibodies against indicated proteins. Membranes were reprobed with β-actin as a loading control. Blots shown are representative of at least three independent experiments. * indicates the same β-actin used for p21 and p27 in MDA-MB-231 cells as the membrane was stripped and reprobed with β-actin antibody after p21 and p27 antibodies. **c**, **d** BrdU uptake in MDA-MB-231 (**c**) and MDA-MB-468 (**d**) cells treated with indicated doses of CB for 24 h followed by BrdU labeling for 4 h. BrdU uptake into cells was detected using anti-BrdU antibody followed by HRP-linked secondary antibody and TMB substrate by colorimetric ELISA. **e** Annexin V-positive MDA-MB-231 and MCF7 cells after treatment with vehicle or CB (10 µM) for 24 h. **f** Western blots of MDA-231 and MCF7 cells treated with vehicle or CB (5 and 10 µM) using antibodies against indicated proteins. Membranes were reprobed with β-actin as a loading control. Blots shown are representative of at least three independent experiments. Data in **a**, **c**, **d**, and **e** are mean ± SEM of three independent experiments. *p* values were calculated using standard Student *t*-tests. *p < 0.05; **p < 0.01; ***p < 0.001; ****p < 0.0001.

Because several CB target genes (including replication licensing factors such as *CDT1, CDC6*, and *MCM-2/4*) play critical roles in cell cycle progression, we examined whether CB affects specific phases of cell cycle progression. CB treatment resulted in G1-phase arrest compared to control in both ER + and TNBC cells as revealed by flow cytometric analysis (Fig. 6a and Supplementary Fig. 23 (gating strategy)). Accumulation of cells in the G1-phase was accompanied by concomitant decreases in the S and G2 phases. Consistent with these results, CB inhibited several G1-specific markers, including cyclin D1, cyclin E, and cyclin-dependent kinase (cdk) 2 and 4, while increasing the level of G1/S transition inhibitor p21 and p27 (Fig. 6b and Supplementary Fig. 24 (unprocessed blots)). Since CB induced cell cycle arrest and inhibited cancer cell viability, we wondered whether it may affect proliferation and/or induce apoptosis. To address the effect of CB on cancer cell proliferation, we treated vehicle and CB-treated cells with 5-Bromo-2′-deoxyuridine (BrdU), a thymidine analog that is incorporated into newly synthesized DNA, and quantified BrdU incorporation using ELISA. CB treatment resulted in significantly reduced BrdU incorporation in a dose-dependent manner compared to vehicle-treated cells (Fig. 6c, d). Next, we examined the effect of CB on apoptosis. Breast cancer cells treated with CB showed cleaved PARP; increased levels of proapoptotic protein BIM; and increased annexin V staining compared with vehicle-treated cells, as revealed by FACS (Fig. 6e) and western blots analysis. (Fig. 6f and Supplementary Figs. 25, 26, 27 (unprocessed blots and gating strategy)).

**Fig. 7:**
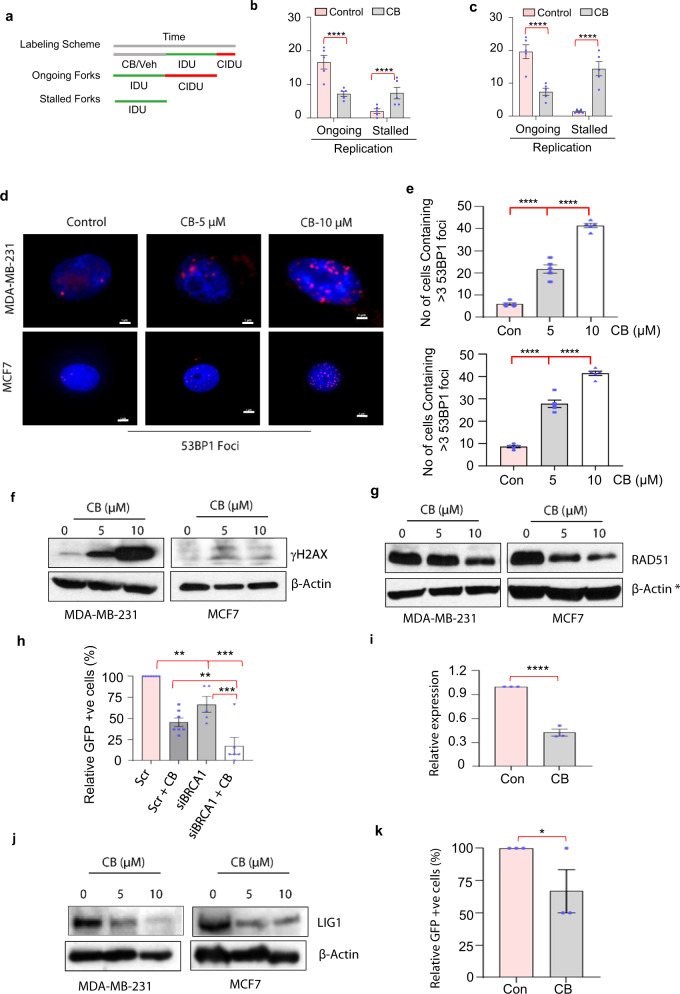
CB blocks replication and inhibits the ability of breast cancer cells to repair DNA by homologous recombination. **a** Schematic for labeling scheme and timing of different replication events. **b**, **c** Percentage of IdU-labeled cells in vehicle (control) and CB-treated MDA-MB-231 (**b**) and HCC1937 (**c**) cells. Cells were pulse-labeled with IdU and CldU sequentially and lengths of replicated tract for progressing fibers were measured by DNA spreading and immunostaining. **d** Immunofluorescence analysis using the antibody against 53BP1 on vehicle control or CB (5 and 10 µM) treated MDA-MB-231 and MCF7 cells. Scale bar, (100X, 1 µm). **e** Average number of cells stained positive for 53BP1 foci in vehicle- or CB-treated MDA-MB-231 (top) and MCF7 (bottom) cells. **f**, **g** Western blot analysis using antibodies against γH2AX (**f**) and RAD51 (**g**) in vehicle (shown as 0)- or CB-treated MDA-MB 231 and MCF7) cells. β-actin was used as a loading control. **h** Flow cytometry analysis showing levels of GFP-positive cells reflecting homologous recombination events in^24^ cells transfected with scrambled or BRCA1-siRNA and treated with vehicle^24^- and CB-treated. DR-GFP integrated U2OS cells were transfected with scrambled or BRCA1-siRNA and treated with vehicle or CB (1 µM) for 12 h, followed by infection with a pCAGGS vector with I-SceI/GFP. GFP + cells and homologous recombination events were determined by flow cytometry after 72 h. The experiment was performed in triplicate along with appropriate control. **i** Real-time PCR analysis on RNA isolated from MDA-MB-231 cells treated with vehicle or CB using LIG1-specific primers. Relative expression of LIG1 was quantified by measuring Ct values and normalized with GAPDH. Results are shown as mean ± SEM for at least three independent experiments. *p* values were calculated using standard Student *t*-tests. ****P < 0.0001. **j** Western blot analysis using antibodies against LIG1 in vehicle (shown as 0)- or CB-treated MDA-MB-231 and MCF7 cells. β-actin was used as a loading control. **k** Flow cytometry analysis showing levels of GFP-positive cells reflecting alt-NHEJ events in cells treated with vehicle or CB. EJ2-GFP integrated U2OS cells were treated with vehicle or CB (1 µM) for 12 h, followed by infection with a pCAGGS vector with I-SceI/GFP. GFP + cells and alt-NHEJ events were determined by flow cytometry after 72 h. The experiment was performed in triplicate along with appropriate control. Data shown as mean ± SEM. *p* value calculated using standard Student *t*-tests. *p < 0.05; **p < 0.01; ***p < 0.001; ****p < 0.0001. * symbols next to β-actin indicate the same loading control as in Fig. 5d.

CB’s inhibition of replication-associated proteins and decreased number of CB-treated cells in the S-phase prompted us to directly examine whether CB may affect replication. To address that question, we performed DNA fiber analysis. We pulse-labeled control and CB-treated MDA-MB-231, breast cancer cells with IdU, washed, pulse-labeled with CldU, fixed cells, and incubated them with IdU (green) and CldU primary antibodies (Fig. 7a). CB treatment inhibited the rate of replication and induced fork stalling and termination (Fig. 7b). In addition to MDA-MB-231, we performed a fiber assay in the BRCA1 mutant HCC1937 cell line to determine whether the change in the status of BRCA1, which regulates replication fork processing^18^ may affect the effect of CB on replication. CB treatment had a more pronounced effect on the fork stalling and termination in HCC1937 cells compared to MDA-MB-231 cells (Fig. 7c and Supplementary Fig. 11a). As replication fork stalling and termination lead to DNA damage and double-strand breaks, we wondered whether CB may directly/indirectly induce DNA damage. To address this, we determined the levels of p53 binding protein (53BP1), which is recruited to the site of DNA damage. Immunofluorescence analysis showed significantly increased numbers of 53BP1 foci in CB-treated breast cancer cells compared with vehicle-treated breast cancer cells (Fig. 7d, e). Further supporting this result, CB treatment resulted in increased accumulation of phosphor γ-H2AX, a marker of double-strand breaks, in breast cancer cells (Fig.7f and Supplementary Figs. 11b,28, 29 (unprocessed blots)).

Because CB induces apoptosis in cancer cells, we reasoned that CB-induced DNA strand breaks may not be properly repaired. To address this issue, we assessed the kinetics of repair by examining levels of RAD51, which is recruited to the site of double-strand breaks to repair damaged DNA during the S/G2 and G1 phases. CB inhibited the RAD51 levels in breast cancer cells (Fig. 7g and Supplementary Fig. 11c). Since RAD51 is highly expressed in breast cancer cells and plays a critical role in homologous recombination^6^-mediated DNA repair, we examined whether HR may also be impaired in CB-treated cancer cells. I-SceI-based GFP assays revealed significantly fewer GFP-positive cells in CB-treated breast cancer cells, suggesting that CB inhibits HR-mediated DNA repair events (Fig. 7h and Supplementary Fig. 11d). Next, we wondered whether CB may interact with BRCA1-mediated DNA repair. To test that, we silenced BRCA1 and performed I-SceI-based GFP assays. CB treatment led to significantly fewer GFP-positive cells in BRCA1-depleted cells compared to scrambled suggesting that CB may have a more pronounced antitumor effect in BRCA1 mutant tumors (Fig. 7h). Since TNBCs with BRCA1 deficiency or ER-positive breast cancers employ alternative DNA repair pathways such as alternative non-homologous end-joining (alt-NHEJ) to repair their DNA and survive/proliferate, we wondered whether, in addition to HR, CB may affect alt-NHEJ. Inspection of our RNA-seq data revealed that the level of LIG1, which supports alt-NHEJ-mediated DNA repair is significantly lower in CB-treated cells compared to vehicle-treated cells. The RNA-seq data was further validated in the real-time PCR and western blot analysis (Fig. 7i, j and Supplementary Fig. 30 (unprocessed blots)). To further confirm these results, we performed an I-SceI-based GFP functional assay. CB inhibited the GFP-positive cells (Fig. 7k and Supplementary Figs. 11e,  31 (gating strategy)) suggesting that CB may inhibit both heightened HR and alternative DNA repair pathways in cancer cells.

**Fig. 8:**
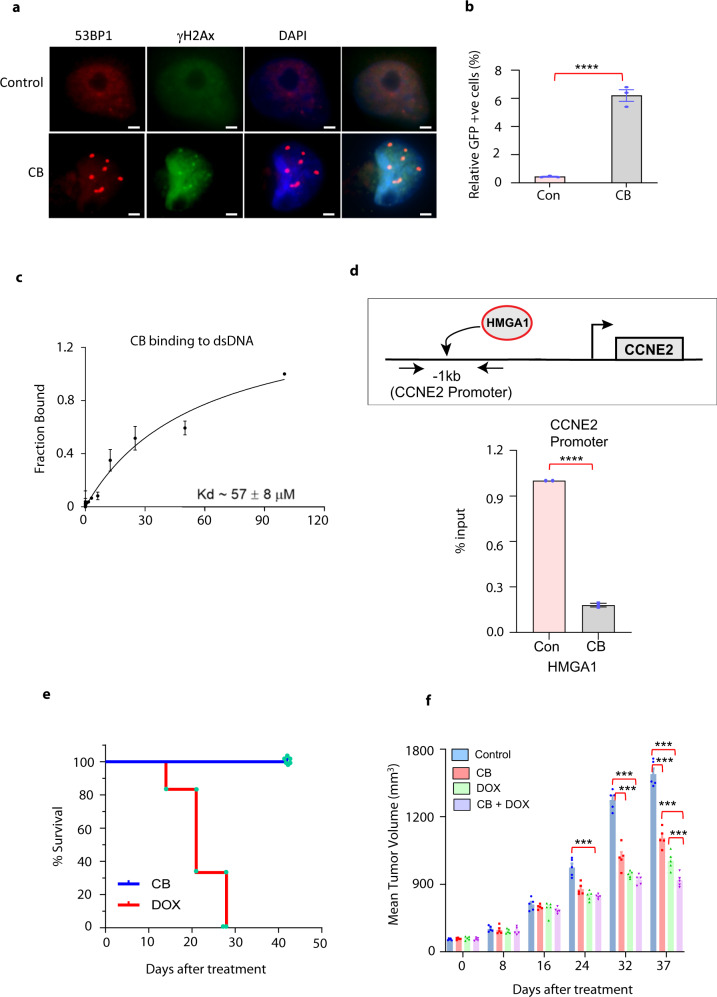
CB is a safer DNA-binding compound. **a** Immunofluorescence analysis using antibodies against 53BP1 and γ-H2AX (green) on vehicle control or CB-treated MDA-MB-231 cells shows co-localization of 53BP1 and γ-H2AX foci. Scale bar, (100X, 1 µm). **b** Flow cytometry analysis showing levels of GFP-positive cells reflecting NHEJ events in cells treated with vehicle or CB. EJ5-GFP integrated U2OS cells were treated with vehicle or CB (1 µM) for 12 h, followed by infection with a pCAGGS vector with I-SceI/GFP. GFP + cells reflecting NHEJ events were determined by flow cytometry after 72 h. The experiment was performed in triplicate along with appropriate control. **c** Fluorescence polarization-based assay showing CB’s ability to bind to double-stranded DNA. **d** Top, schematic illustration of chromatin immunoprecipitation (ChIP) primers flanking the HMGA1 binding site in the promoter region of the human cyclin E2 gene. Bottom, ChIP-qPCR showing fold change in the recruitment of HMGA1 to the cyclin E2 promoter in vehicle or CB (1 µM)-treated MDA-MB-231 cells. **e** Survival rate of mice treated with vehicle, CB (3 mg/kg body weight), or DOX (2.5 mg/kg body weight) conjugated with intralipid every 5 days (*n* = 6). **f** Mean tumor volume in vehicle- and CB conjugated with intralipd + doxorubicin (Dox)-treated mice. MDA-MB-231 cells were subcutaneously implanted into mammary fat pads of athymic nude mice. After tumors reached approximately 100–150 mm^3^, mice were treated with vehicle, CB (1.5 mg/kg bw), DOX (1 mg/kg bw) conjugated with intralipid and CB (1.5 mg/kg bw)-intralipid + DOX (1 mg/kg bw) combination every 5 days for 30 days (*n* = 6). Mice were kept under observation without treatment for one more week before sacrificing. *p* value calculated using standard Student *t*-tests. ***p < 0.001; ****p < 0.0001.

Our results showed that CB treatment increased the p53BP1 and γ-H2AX foci in breast cancer cells. Since p53BP1 generally accumulates during NHEJ, we wondered whether CB treatment may result in increased NHEJ to compensate for the loss of HR in breast cancer cells. To address that, first, we performed p53BP1 and γ-H2AX co-localization studies. Immunofluorescence analysis showed increased p53BP1 and γ-H2AX co-localization in CB-treated cells compared to vehicle-treated cells (Fig. 8a). Next, we measured NHEJ-mediated DNA repair by performing an I-SceI-based EJ5-GFP functional assay. CB treatment resulted in a significantly increased number of GFP-positive cells compared to vehicle-treated cells suggesting a shift towards classical NHEJ-mediated DNA repair following CB treatment (Fig. 8b and Supplementary Fig. 11f). These observations along with increased apoptosis following CB treatment suggest that NHEJ-mediated repair is not robust enough to compensate for the loss of HR and/or alt-NHEJ in CB-treated breast cancer cells.

### Mechanism of CB target gene regulation

CB’s inhibition of replication and DNA repair prompted us to examine whether CB may bind to DNA, as does its parent compound carbazole^19^. To address this question, we first performed fluorescence polarization (FP) and UV-Vis absorbance assays. For FP, a 21-nucleotide double-stranded DNA oligo was synthesized with a fluorescein moiety attached to the 5′-end of the top DNA strand. Fluorescence polarization changed significantly when 10 nM of fluorescein-labeled DNA was incubated with increasing concentrations of CB in a cell-free system (Fig. 8c). CB binds to DNA with a dissociation constant (*K*_d_) of ~57 μM. To begin to address the specific mechanism/s by which CB may regulate its target gene expression, we decided to focus on transcription factor/s that binds to AT-rich DNA region/sequences like CB’s parent compound carbazole. We focused on HMGA1, a transcription factor that is known to bind to AT-rich sequences/regions and transactivate several cell cycle and DNA repair genes that showed reduced expression in CB-treated breast cancer cells. Moreover, HMGA1 is known to support the growth and progression of breast cancers. Our RNA-seq data suggested that *HMGA1* may not be the direct target of CB as there was no difference in the expression of *HMGA1* between the vehicle or CB-treated cells. Next, we tested whether CB may indirectly affect the activity of HMGA1. To do that, we performed ChIP analysis on vehicle and CB-treated breast cancer cells using primers spanning the promoter regions of *cyclin E*, which is a known target of HMGA1 and showed reduced expression in CB-treated breast cancer cells. ChIP analysis on CB-treated MDA-MB-231 cells showed significantly reduced recruitment of HMGA1 on the promoter of cyclin E compared to vehicle-treated cells (Fig. 8d). These results indicate that CB may bind to cyclin E promoter and block HMGA1 recruitment and consequently transactivation of cyclin E.

### CB is a more potent and safe drug than other chemotherapy agents

Some currently used cancer treatment drugs target DNA to kill tumor cells, such as doxorubicin and cisplatin^20, 21^. We compared the safety profiles of CB and doxorubicin. Doxorubicin (2.5 mg/kg bw; once a week) treatment at a dose lower than CB (3 mg/kg bw) resulted in 100% death of tumor-bearing mice by day 25, while CB treatment had no effect on the survival of mice until termination of the study at day 50 (Fig. 8e). Since CB binds to DNA and affects DNA damage repair events, we wondered whether CB may improve the efficacy of DNA-targeting chemotherapy drugs like doxorubicin. Cell viability assays using sub-optimal doses (IC_20_) of CB and doxorubicin showed the combination synergistically inhibited breast cancer growth compared to the effects of either agent alone (Supplementary Fig. 11g). To further validate these results, we performed in vivo tumor xenograft studies using doxorubicin at a sub-optimal dose that does not affect the survival of the mice. Our results revealed that a combination of CB with doxorubicin had significantly reduced tumor growth compared to either CB or doxorubicin alone (Fig. 8f). These results indicate that CB may be a safe and potent therapeutic adjuvant for treating breast cancers (Supplementary Fig. 12).

## Discussion

Our study indicates that CB has the potential to be a safe and potent therapeutic regimen for treating cancer in general and breast cancers in particular. Using physiologically relevant preclinical models, including patient-derived xenografts and ex vivo explant models, we showed that CB inhibited TNBC, ER+, and ER mutant breast cancer growth and metastasis without targeting normal mammary tissue. We also showed that CB inhibits breast cancer growth and metastasis by binding to DNA and inhibiting uncontrolled replication and DNA repair pathways that breast cancer cells use to survive.

Although a deficiency in some aspects of the DNA repair pathway is a hallmark of cancers including breast cancers, cancer cells survive and meet the demands of incessant replication by repairing (albeit with errors) their DNA, presumably by alternative DNA repair pathways^22^. For example, breast cancer cells with deficiencies in HR proteins (such as BRCA1) can repair their DNA by either relying on other highly expressed HR-related proteins (such as RAD51 or PARP1) or backup DNA repair pathways such as alt-NHEJ^23^. Our results showing inhibition of HR-mediated DNA repair pathways by CB suggest that CB may inhibit DNA repair pathways that cancer cells rely on to support their growth. Moreover, these alternative DNA repair pathways may not be the predominant DNA repair pathways used by normal cells; this may be one reason for the negligible toxicity observed in CB-treated mice and normal cells.

The alteration of cellular DNA and the dependency of cancer cells on incessant replication were the initial reasons for targeting DNA as cancer therapy^24^. However, DNA-targeting drugs used in clinics today have major limitations, including life-threatening toxicity^20, 21^. These problems mostly stem from the ability of DNA-targeting drugs to indiscriminately bind to cellular DNA or other non-DNA macromolecules, resulting in DNA damage^25^. We reason that drugs with high sequence/region specificity that do not directly damage the DNA and take advantage of DNA-related processes that tumor cells (but not normal cells) use could have favorable therapeutic outcomes. Based on our results, it is likely that CB may inhibit cancer growth and metastasis by targeting specific DNA sequences and regions critical for the survival of cancer cells.

AT-rich scaffold/matrix attachment regions (S/MARs) could be one such region. The S/MARs function in several processes, including increasing the rate of transcription initiation, providing support to the origin of replication, and consequently facilitating replication^26^. Furthermore, several S/MAR/BUR-binding proteins, including SATB1, HMGA1, p114, PARP1, nucleolin, and mutant p53, are overexpressed and support the growth and progression of breast cancers^26–31^. Our results showing CB-induced replication fork stalling suggest that CB may affect S/MAR function in cancer cells. CB’s parent compound, carbazole, binds to AT-rich regions on DNA, so there is some biological validity to this notion.

CB’s targeting of specific DNA regions and related functions critical for cancer cell survival but dispensable for normal cells may be one reason that CB has negligible toxicity towards normal cells. For example, CB may block regulatory elements in the promoter or enhancer regions that are critical for the expression and activity of CB target genes involved in the cell cycle and DNA repair machinery, which are uniquely expressed in highly proliferative cancer cells^1, 4^. Consistent with that, we show significantly reduced enrichment of HMGA1 on cyclin E promoter in CB-treated breast cancer cells. In addition, CB may inhibit the expression of specific targets of pro-oncogenic proteins that support the growth and progression of cancer in general and breast cancers in particular. It is likely that CB interference with critical elements essential for cancer cell proliferation makes it several orders of magnitude more lethal to cancers and safer than drugs that cause less region-specific DNA damage^21^. However, further detailed DNA-binding studies are warranted to determine CB’s specificity to S/MARs regions of the genome.

In summary, CB inhibits tumor growth and metastasis by targeting cell cycle, replication, and DNA repair events. In addition, we show that CB may be equally effective against therapy-resistant ER-positive breast cancers. Furthermore, we show that CB may improve the efficacy of DNA-targeting chemotherapy drugs for treating breast cancers. Our work suggests that CB’s potent antitumor effects and negligible toxicity may be due to its binding to specific sequences of DNA. Collectively, our results indicate that CB is a safe and potent therapeutic with immense translational potential, and provide a strong rationale for its development for rapid clinical testing.

## Methods

### Synthesis of CB

CB was synthesized using a protocol modified from^32^. Briefly, 1.0 g carbazole (Sigma) was added to 1.02 g of 4,4′-diethylaminobenzophenone (Fisher Scientific) under argon at room temperature. Phosphorus oxychloride was added (~5 ml) and heated at 107 °C for 4 h. The reaction was quenched with water and the solvent was removed via rotary evaporation at 35 °C. The resulting solid was extracted in chloroform. Chromatographic separation over Alumina with 100:1 ethyl acetate:hexane was used to remove excess 4,4′-diethylaminobenzophenone. Finally, extraction with 50:50 chloroform:methanol produced the CB dye in 95% yield as a metallic blue solid. High-resolution mass spectrometry was performed by the Mass Spectrometry Center of UTHSCSA showing a compound with: 588.34 (M+). Absorbance was determined using a spectrophotometric plate reader (Bio-Tek) using a range of wavelengths from 200 to 800 nm. Background (ethanol) was subtracted out to yield the final curve, giving a maximum absorbance peak of 610 nm. Nuclear magnetic resonance analysis was performed as described in refs. ^32, 33^.

### Human breast cancer cell lines and culture conditions

The breast cancer cell lines MDA-MB-231, MDA-MB-468, BT-549, MCF7, ZR-751, and SKBR3 were purchased from ATCC (Manassas, VA) and cultured in DMEM supplemented with 10% fetal bovine serum, penicillin (100 U/ml) and streptomycin (100 µg/ml) in a humidified 5% CO_2_ incubator at 37 °C.

### Breast cancer tissues

For ex vivo explants, de-identified breast cancer tissues from five patients (three triple-negative breast cancer and two estrogen receptor-positive) along with normal matched tissues were collected from the Breast Cancer Clinic at UT Health Science Center San Antonio, after obtaining approval from Institutional Review Board, UT Health San Antonio (IRB #HSC20120041H). All relevant ethical regulations were followed before collecting the tissues.

### Cell proliferation assays

Breast cancer cells were seeded in 96-well plates at a density of 5 × 10^3^ cells/well and after 20–24 h of incubation, cells were treated either with DMSO alone (0.02%, vehicle control) or with varying concentrations of CB (0.5–20 µM) in DMSO for an additional 24, 48, and 72 h in a CO_2_ incubator at 37 °C. For Nano-CB cell viability, CB is mixed with 20% intralipid at desired concentrations. Cell viability was assessed by using CellTiter-Glo assays (Promega Inc.). For combination studies, MDA-MB-231 cells are treated with vehicle, low dose of CB (200 nM), DOX (5 nM), and CB + DOX combination for 72 h. Cell viability was assessed by using CellTiter-Glo assays (Promega Inc.).

### Colony formation assays

About 200,000 cells per well were plated in six-well plates and after 20–24 h of incubation, cells were treated either with DMSO alone or with varying concentrations of CB (1–10 µM) in DMSO for another 24 h. Next, 1000 cells/well were re-seeded in six-well plates for an additional 7 days until colonies were clearly visible. Colonies were fixed with 4% paraformaldehyde and visualized by staining with 1% crystal violet and wells were scanned using a scanner. Visible colonies were counted using ImageJ software.

### Invasion and migration assays

Breast cancer cells were pretreated with CB at different concentrations for 24 h. About 25,000 cells were added to the top chamber of the transwell and plates were placed in a 5% CO_2_ incubator overnight. For invasion assay, cells were added to the top well coated with the matrigel of the invasion chamber. Migrated/invaded cells were fixed with 4% paraformaldehyde, and stained with 0.5% crystal violet. Migrated/invaded cells were visualized and counted under the microscope^15^.

### Pharmacokinetics

CB plasma concentrations were measured using a validated liquid chromatography-tandem mass spectrometry method as previously described^34^. Three to four Balb/C mice per treatment timepoint were injected intraperitoneally with CB (3 mg/kg bw), and plasma was collected at 0, 15, 30, 60, 120 min, and 24 h post-treatment.

### Animal studies

All animal experiments were performed after the protocol was approved by the UTHSCSA Institutional Animal Care and Use Committee. Mice were housed in accordance with UTHSCSA’s protocols for animal experiments and in keeping with established guidelines. For orthotopic xenograft tumor assays, 2 × 10^6^ MDA-MB-231 cells were mixed with an equal volume of Matrigel™ and implanted in mammary fat pads of 6-week-old female athymic nude mice, as previously described^15, 35^. Once tumors reached 100–150 mm^3^ in size, mice were randomly divided into control and treatment groups. Group 1 served as controls and received vehicle (DMSO). Groups 2 and 3 received CB conjugated with intralipid (1.5 and 3 mg/kg/body weight in 20% intralipid) intravenously once a week for 4 weeks, respectively. For the combination studies, mice were randomly divided into vehicle, CB-intralipid alone (1.5 mg/kg body weight), doxorubicin alone (1 mg/kg body weight), and CB-intralipid + doxorubicin treatment groups. Tumor volumes and body weight were measured twice a week. After 4 weeks of treatment, mice were euthanized, and tumors were isolated and processed for molecular and immunohistological studies. Tumor volume was calculated by using the formula 0.5236*L*_1_ (*L*_2_)^2^, where *L*_1_ is the long axis and *L*_2_ is the short axis of the tumor. At the end of the experiment, mice were sacrificed, and tumors were excised, weighed, and fixed in buffered formalin for further analysis.

### PDX model

These studies were approved by the Baylor College of Medicine Institutional Animal Care and Use Committee. Small tumor pieces (3-mm^3^) from triple-negative breast cancer patients (BCM-4013) were transplanted into the fourth mammary fat pads of 5 to 6 weeks old SCID/Beige female mice^36^. When the tumor volumes reached ~100 mm^3^ 50-days after transplantation, mice were treated with vehicle or CB (3 mg/kg body weight, intraperitoneally) every four days. Tumor volumes were measured every 6 days. After 9 weeks of treatment, the mice were euthanized, and the tumors were isolated and processed for molecular and immunohistologic studies. Tumor volume was calculated by using the formula 0.5 × *L* × *W* × *H*, where *L* is the length, W is the width, and *H* is the height of the tumor.

### Short and long term toxicity studies

Short and long-term toxicity studies were carried out in immunocompetent BALB/cJ mice. BALB/cJ mice received either received vehicle (control) or nano-CB (3 mg/kg b.w. intravenously) once a week for 4 weeks. To evaluate short-term toxicity, one set of mice from both control and CB-treated groups (*n* = 4) was sacrificed within 24 h of the final CB dose. For long-term toxicity studies, mice were followed for an additional 2 months. At the end of the experiment, mice were sacrificed and observed for visible toxicity and lung, liver, kidney, and spleen were excised and fixed in buffered formalin for further analysis.

### Ex-vivo explants

For patient-derived explants, excised breast tumor and normal adjacent matched tissues from triple-negative and ER^+^ breast cancer patients were provided by a pathologist in accordance with an IRB-approved protocol at UTHSCSA (Control# HSC20120041H). Small tumor and normal adjacent tissue pieces were dissected into multiple 1 mm^3^ pieces and cultured on a pre-soaked gelatin sponge (Johnson and Johnson, Brunswick, NJ) in 500 μl RPMI supplemented with 10% fetal bovine serum, 1% antibiotic/anti-mycotic solution, 0.01 mg/ml hydrocortisone, and 0.01 mg/ml insulin. Vehicle or nanoparticle conjugated-CB 5 µM were added to the media-containing tissues and kept at 37 °C in a 5% CO_2_ incubator for 72 h. Explant tissues were subsequently either formalin-fixed and paraffin-embedded or preserved for RNA isolation as previously described^15^. For ER mutant WHIM20-ER + Y537S PDX explant, ER mutant WHIM20-ER + Y537S PDX were purchased from Horizon Discovery Ltd and were initially established in SCID mice as described previously^37^. When the tumor reached 1000 mm^3^, they were dissected into 2-mm cubes. Tumor samples were incubated on gelatin sponges for 24 h in culture followed by treatment with either vehicle, CB (10 μM) for 72 h. Representative tissues of ER mutant PDX explants were fixed in 10% formalin at 4  °C overnight and subsequently processed into paraffin blocks. The sections were then processed for immunohistochemical analysis.

### Ki67 and TUNEL analyses

For immunohistochemical analyses, explants and xenograft tumor tissues were processed, paraffin-embedded, and incubated with an antibody against Ki67 (#NB500, Novus, 1:100) or subjected to apoptosis assay using a TUNEL assay kit (#G3250, Promega Inc.) as described previously^15^. Ki67 and TUNEL-positive cells were counted at ten arbitrarily selected fields at 40X magnification. The proliferation/apoptotic index (per 40X microscopic field) was determined as (number of Ki67/TUNEL-positive cells × 100)/total number of cells.

### Gene expression profiling

Total RNA was isolated from MDA-MB-231 cells following treatment with vehicle and CB for 24 h, respectively. RNA samples were further processed at the UTHSCSA Genomics Core for gene expression profiling using an Illumina Human HT-12 v4 Expression BeadChip following the manufacturer’s standard protocol (Illumina, San Diego, CA). Gene expression data were quantified and normalized (quantile normalization) using BeadStudio software (Illumina). We used the LIMMA package to perform differential gene expression analysis (R/Bioconductor)^38^, where samples are the first quantile normalized and then LIMMA fits a linear model to the expression data for each probe and evaluates the differential expression with a moderated t-statistic by applying the Empirical Bayes method and shrinking the standard errors towards a common value. The averaged mean expression level for each test group, log2 fold change, *p* value, and multiple test adjusted *p* value were reported for each gene, and significantly differentially expressed genes were selected based on (1) fold change >2, (2) mean expression >7, and the multiple test adj *p* value <0.05. Raw data have been deposited in Gene Expression Omnibus (GSE161911).

### RNA and protein analyses

Total RNA from cell lines, PDX tumors, and explants was extracted using miRNeasy kit (Qiagen Inc. Cat.No: 217004) and subjected to qRT-PCR using iScript cDNA synthesis kit (Biorad Inc. Cat. No:1708891) and iTaq Universal SYBR Green Supermix (Biorad Inc; Cat. No: 1725124). Western blot from vehicle and CB-treated cell lines and tumors were performed using standard protocol^15, 39, 40^. Table 2 lists primer sequences for all genes. Supplementary table 1 lists the antibody information for all proteins used in the present study. Primary antibody dilutions are as follows: CDC6, CDT1, Claspin, MCM2, MCM4, CDC25, RRM1, RRM2, Cyclin D1, CyclinB1, Cyclin E1 (1:500). CDK2, CDK4, PARP1, Phospho γ-H2AX, RAD51, p21, P27, BIM, Lig 1 (1,1000), and β-Actin (1:50,000). Secondary HRP conjugated antibodies were used at 1:5000 dilution.

**Table 2:** List of primers used in the study.

S.No	Gene	Primer sequence	
1.	CDC6	Forward	5′ -GGAGATGTTCGCAAAGCACTGG -3′
		Reverse	5′-GGAATCAGAGGCTCAGAAGGTG -3′
2.	CDT1	Forward	5′ -AGGACACCATCTCTGAGCTTG -3′
		Reverse	5′ -GCACCTGGTACTTGTAGGGC -3′
3.	MCM2	Forward	5′- ATGGCGGAATCATCGGAATCC-3′
		Reverse	5′ – GGTGAGGGCATCAGTACGC-3′
4.	MCM4	Forward	5′- GACGTAGAGGCGAGGATTCC -3′
		Reverse	5′- GCTGGGAGTGCCGTATGTC -3′
5.	CCND1	Forward	5′- GTGCTGCGAAGTGGAAACC-3′
		Reverse	5′- ATCCAGGTGGCGACGATCT-3′
6.	WEE1	Forward	5’- ATTTCTCTGCGTGGGCAGAAG-3′
		Reverse	5′- CAAAAGGAGATCCTTCAACTCTGC-3′
7.	ATMIN	Forward	5′CAACCAATCCCTAGACCAGACA-3′
		Reverse	5′GCATCACGGGTAGTTTAACCAAA-3′
8.	GINS2	Forward	5′-CCAATGCCCAGCCCTTACTAC-3′
		Reverse	5′-CTGCCTTCGGGATGTTGTCT-3′
9.	GINS4	Forward	5′-AGTTGGCCTTTGCCAGAGAG-3′
		Reverse	5′-GAACTGCCCGAAAGAGGTCC-3′
10.	GAPDH	Forward	5′-GGGTGTGAACCATGAGAAG-3′
		Reverse	5′-GACTGTGGTCATGAGTCCT-3′
11.	18 S	Forward	5′-CGGACCAGAGCGAAAGCAT-3′
		Reverse	5′-CCTCCGACTTTCGTTCTTGATT-3′

### DNA fiber analysis

For DNA fiber analysis, MDA-MB-231 cells were treated with vehicle or CB for 36 h before being pulse-labeled with IDU followed by CIDU^41^. DNA fibers were spread on slides, incubated with 2.5 M HCl, and washed with PBS followed by blocking with 2% BSA in PBS. Primary antibody in blocking buffer was added to the slides for 1 h followed by multiple washes. A secondary antibody was applied for 1 h and slides were mounted with Vectashield mounting medium. Fibers were analyzed under the microscope. Pictures were taken from randomly selected fields from both vehicle and CB-treated groups. Images were analyzed using ImageJ software. A minimum of 100 individual fibers were analyzed and the relative frequency of ongoing and stalled forks was scored^41^.

### Immunofluorescence

To determine DNA damage, immunofluorescence was performed with vehicle and CB-treated cells using 53BP1 rabbit antibody (#A300-272AT, Bethyl Laboratories). Breast cancer cells were grown on coverslips and treated vehicle or CB. The cells were then fixed with 4% cold paraformaldehyde followed by permeabilization and blocking using 5% normal goat serum in 0.1% Triton X-100 for 30 min at room temperature. The cells were then incubated with anti-53BP1 antibody (1:200 dilution; Bethyl Laboratories) and Phospho γ-H2AX (1:200 dilution; Ab2893) overnight followed by incubation with TRITC-conjugated goat anti-rabbit secondary antibody (1:500). The cells were counterstained with DAPI and observed under a fluorescence microscope^15, 40^.

### Homologous recombination, non-homologous end-joining (NHEJ), and alternative non-homologous end-joining (Alt-NHEJ) assays

The I-SceI-based DR-GFP^6^, EJ5-GFP (NHEJ), and EJ2-GFP reporter assay was performed in U2OS cells following CB treatment (1 µM, for 24 h) to evaluate the frequency of DNA strand break repair by HR^42, 43^, NHEJ, and alt-NHEJ^44^. U2OS cells stably expressing DR-GFP, EJ5-GFP, and EJ2-GFP were transfected with pCAG or I-SceI expression vector for 24 h, followed by treatment with vehicle or CB. The percent of GFP-positive cells were evaluated by flow cytometry using BD FACSCanto™ II and BD LSRFortessa™ X-20 instruments. Data were analyzed using BD FACSDiva™ software.

### Cell cycle distribution and apoptosis assay by flow cytometry

For cell cycle analysis, breast cancer cells were treated with vehicle or CB and fixed with 70% ethanol for 24 h and stained with propidium iodide. Live cells were subjected to cell cycle distribution using a BD FACSCelesta™ or BD LSRFortessa™ X-20 instruments.

For apoptosis assays, cells were treated with vehicle and CB followed by staining with annexin V/propidium iodide using the ApoAlert™ Annexin V-FITC Apoptosis Kit (Clontech, Cat # 630110). The percent of annexin V-propidium iodide-positive cells were determined using flow cytometry.

### Determining the equilibrium binding affinity of CB to double-stranded (ds) DNA

We measured equilibrium binding using a fluorescence polarization (FP)-based assay. A 21-nucleotide long ds DNA oligo (see the sequence below) was synthesized with a fluorescein moiety attached to the 5′-end of the top DNA strand and purified by the High-performance liquid chromatography (IDT Technology). A constant concentration of fluorescein-labeled DNA (10 nM) was titrated with increasing concentrations of CB (0–100 μM) in a 384-well plate format in a buffer containing 10 mM HEPES pH 7.5, and 50 mM KCl. The fluorescence polarization (emission wavelength = 530 nm, excitation wavelength = 485 nm) value for each dilution was measured using PHERAstar FS (BMG Labtech). Buffer-corrected values of triplicate runs were used to calculate the equilibrium dissociation constant for CB binding to this DNA using a simple 1:1 specific binding model.

The double-stranded DNA (ds) oligo used in this study was:

5′- FAM - ATACAGCAGTAGACTATGATA

3′ TATGTCGTCATCTGATACTAT

### Chromatin immunoprecipitation (ChIP)

MDA-MB-231 cells were treated with vehicle or 1 μM CB for 48 h and then harvested for ChIP assays using a Magna ChIP™ A/G Chromatin Immunoprecipitation Kit (Millipore, Cat #.17-10085). ChIP assays were performed using antibodies against HMGA1 (ab252930, Abcam) and normal IgG antibodies, according to the manufacturer’s protocol. qPCR was performed with primers flanking the cyclin E promoter. Relative fold change of HMGA1 recruitment on cyclin E promoter was determined by calculating the fold enrichment of target antibodies over IgG, followed by normalization of CB-treated samples over the vehicle.

### Statistics and reproducibility

All values and error bars in graphs are means ± SEM; respective *n* values are indicated in Figure legends. *P* values were determined by two-tailed Student’s *t*-tests using Graphpad Prism 8 software. A value of *p* < 0.05 was considered statistically significant. All the results shown in this manuscript were generated using multiple cell lines, animals, and sufficient biological replicates (at least three or more) to attain statistically significant outcomes.

### Reporting Summary

Further information on research design is available in the Nature Research Reporting Summary linked to this article.

## Supplementary information

Supplementary Information

Description of Additional Supplementary Files

Supplementary Data 1

Supplementary Data 2

Reporting Summary

## Data Availability

Raw RNA-seq data have been deposited in Gene Expression Omnibus, accession number GSE161911. Unprocessed blots can be found in Supplementary Figs. 13–28. The source data behind the graphs in the paper can be found in Supplementary Data 1. The source data behind the volcano plot, Ingenuity pathway analysis, and real-time PCR analysis can be found in Supplementary Data 2. All other data are available from the authors on reasonable request.
