# Supplementary material for: Targeting aberrant replication and DNA repair events for treating breast cancers
Source: Commun Biol. 2022 May 24;5:493. doi: 10.1038/s42003-022-03413-w (PMC9130234; doi:10.1038/s42003-022-03413-w)
Supplement: Supplementary file 1 — Supplementary Information [file 42003_2022_3413_MOESM1_ESM.pdf]

Supplementary Table 1: Antibodies used in the present investigation.

S.NO	Antibody	Cat Log No	Source
1.	CDC6	#3387	Cell Signaling Technology
2.	CDT1	Sc-365305	Santacruz Biotechnology
3.	Claspin	#2800S	Cell Signaling Technology
4.	MCM2	Sc-373702	Santacruz Biotechnology
5.	MCM4	Sc-48407	Santacruz Biotechnology
6.	CDC45	#3673	Cell Signaling Technology
7.	RRM1	Ab226391	Abcam
8.	RRM2	Ab57653	Abcam
9.	Cyclin D1	#2978	Cell Signaling Technology
10.	Cyclin B1	#4135	Cell Signaling Technology
11.	CyclinE1	#4129	Cell Signaling Technology
12.	CDK2	#2546	Cell Signaling Technology
13.	CDK4	#12790	Cell Signaling Technology
14.	PARP1	#9532	Cell Signaling Technology
15.	Phospho ɣH2AX	Ab2893	Abcam
16.	53BP1	#A300-272A	Bethyl Laboratories
17.	RAD51	Sc-8349	Santacruz Biotechnology
18.	β-Actin	#A3854	Sigma Aldrich
19	P21	#2947	Cell Signaling Technology
20	P27	#3686	Cell Signaling Technology
21	BIM	#2933	Cell Signaling Technology
24	Lig1	ab177946	Abcam

Supplementary Figure 1

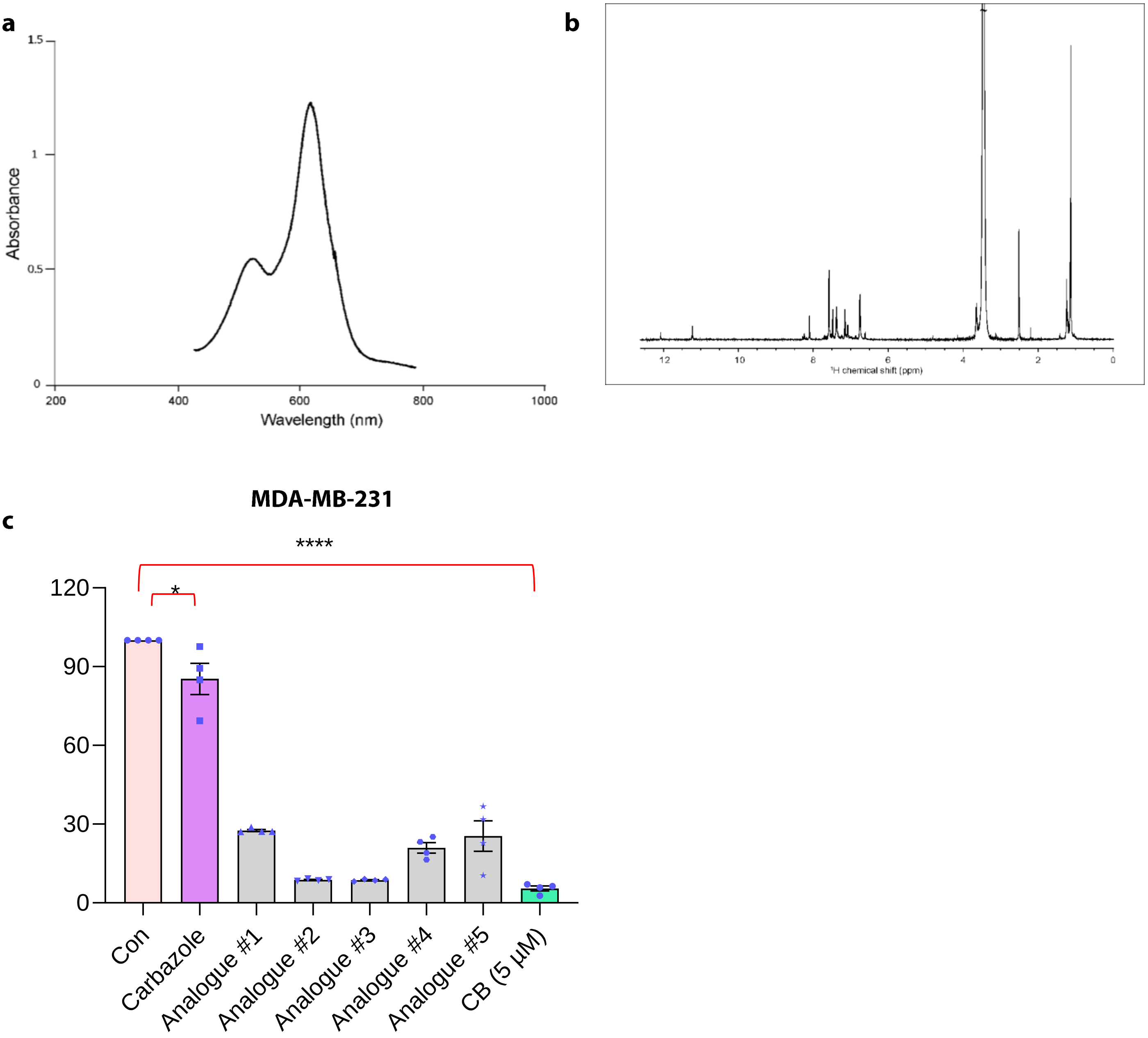

Supplementary Figure 1. Carbazole Blue is a novel anti-cancer small molecule. ***(*a)** Absorbance spectrum of carbazole blue (CB) showing maximum peak at 610 nm. **(b)** NMR spectrum of CB confirms structure. **(c)** MDA-M231 cells treated with vehicle (Con) or CB (5 μM) or other CB analogs for 72 hours. *, $p<0.01$; ****, $p<0.0001$ versus control group, t test; CB-Carbazole blue.

Supplementary Figure 2

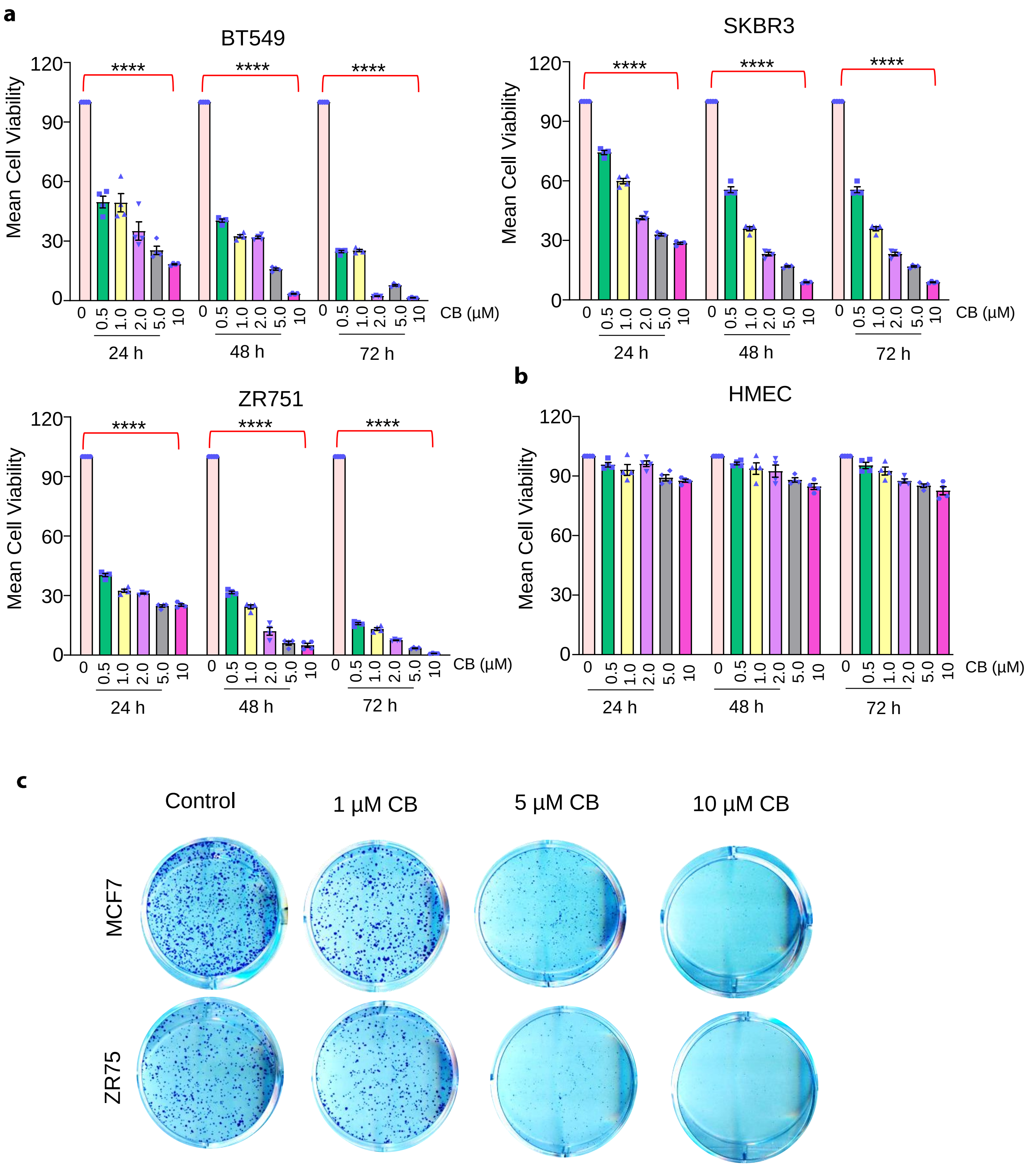

Supplementary Figure 2. CB inhibits breast cancer cells growth without affecting normal mammary cells. BT-549, SKBr3 and ZR751 cells (a) as well as normal mammary epithelial cells (HMEC) (b) were treated with vehicle control (DMSO) or indicated doses of CB (0.5–10 μM) for 24, 48 and 72 hours. Cell viability was assessed using Cell Titer-Glo Luminescent viability assay. (c**)**. MCF-7 and ZR751 cells were seeded in 6-well plate and pre-treated with vehicle and 1, 5 and 10 μM doses of CB for 24 hours. Cells were re-seeded and allowed to grow for additional 7 days and colonies were stained with crystal violet. ****, $p<0.0001$ versus control group, t test; CB-Carbazole blue.

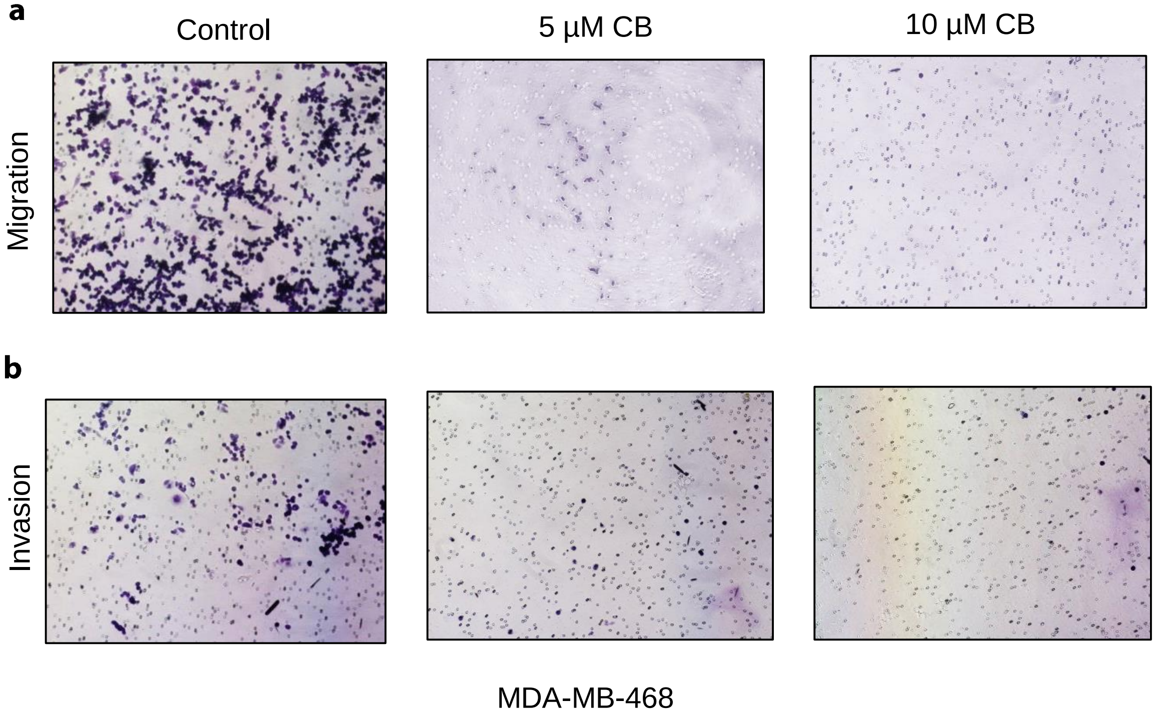

Supplementary Figure 3. CB inhibits migration and invasion of breast cancer cells. Photomicrographs of migrated (a) and invaded (b) MDA-MB-468 cells pre-treated with vehicle or 5 and 10 µM doses of CB. Bar graphs show number of migrated and invaded cells counted microscopically in six different fields per filter. The data shown are mean ± SEM for three independent experiments. CB-Carbazole blue.

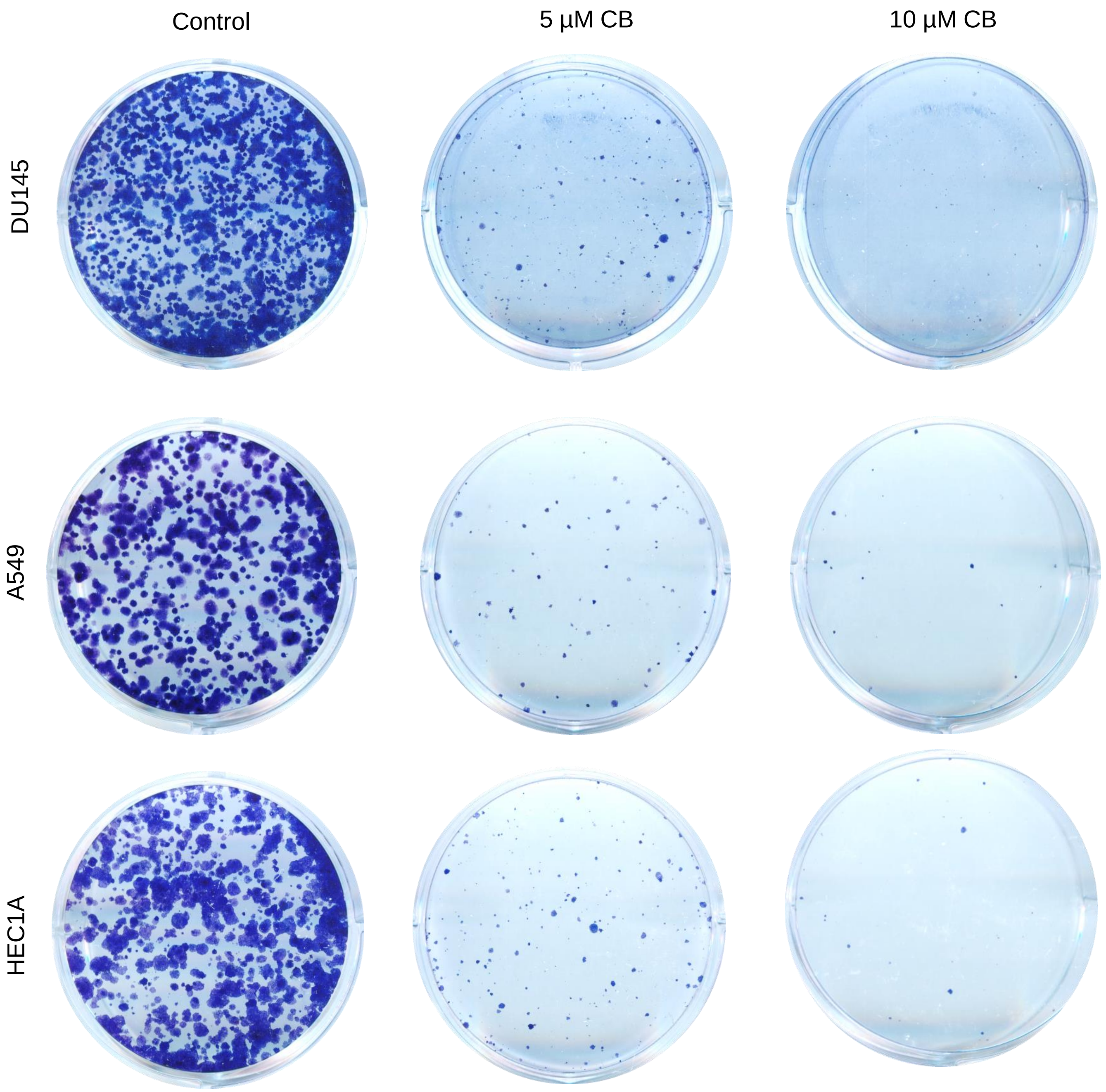

Supplementary Figure 4. CB inhibits prostate, lung and endometrial cancer cells growth. DU145, A549 and HEC1A cells were seeded in 6-well plate and pre-treated with vehicle and 5 and 10 µM doses of CB for 24 hours. Cells were re-seeded and allowed to grow for additional 7 days and colonies were stained with crystal violet. CB-Carbazole blue.

.

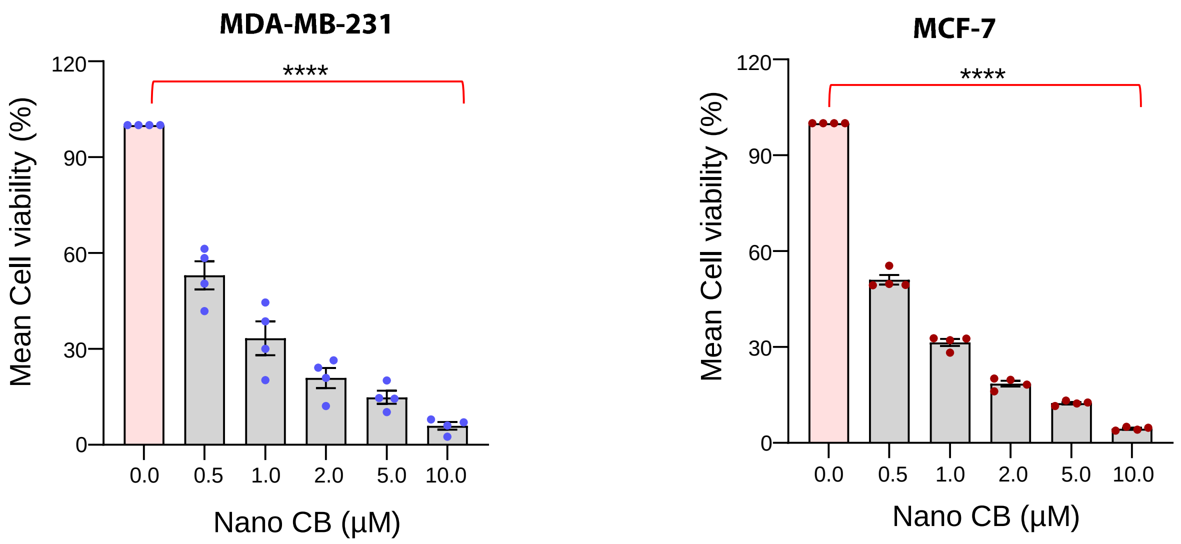

Supplementary Figure 5. Nano CB inhibits breast cancer cells growth. MDA-MB-231 and MCF7 cells were treated with vehicle or indicated doses of CB (0.5–10 μM) conjugated with intralipid (nano CB) for 48 hours. Cell viability was assessed using Cell Titer-Glo Luminescent viability assay. ****, $p<0.0001$ versus control group, t test; CB-Carbazole blue.

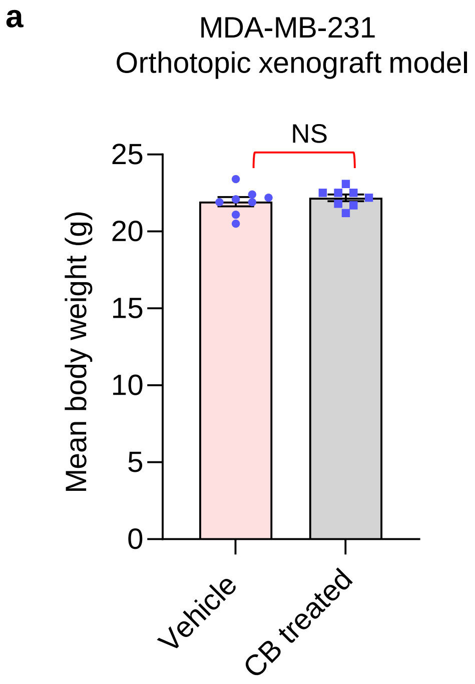

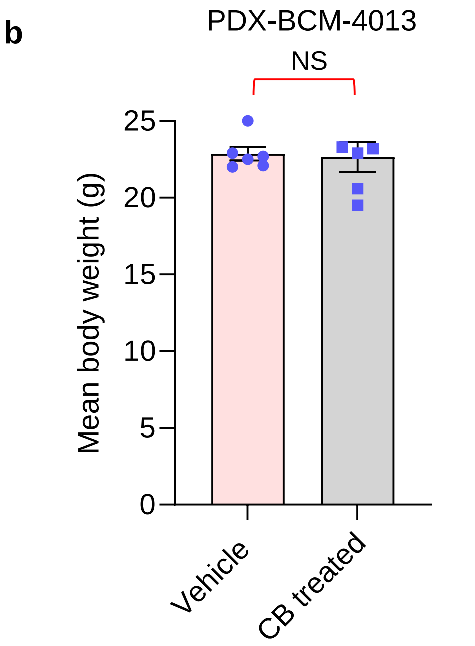

Supplementary Figure 6. Nano CB does not induce any body weight changes in mice. (**A**) Bar graph showing no significant body weight changes in MDA-MB-231-orthotopic xenograft model after 30 days of CB conjugated with intralipid treatment (3 mg/kg bw, every 5 days for 30 days) (mean ± SEM; n=8). (**B**) Bar graph showing no significant body weight changes in BCM-4130 PDX model after 30 days of CB-intralipid treatment (3 mg/kg bw, weekly twice for 98 days) (mean ± SEM; n=6).NS- not significant, t test; CB-Carbazole blue.

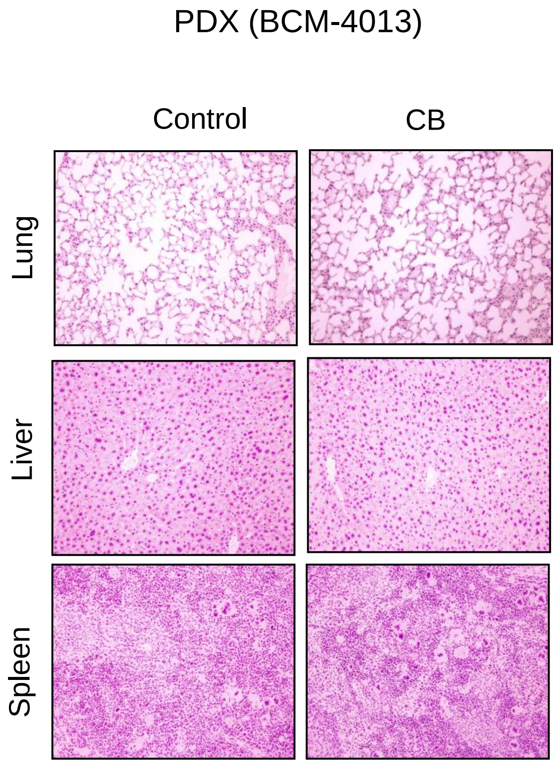

Supplementary Figure 7. CB is a safe and viable anti-cancer compound. Representative H&E stained sections of lung, livers and spleens from nano-CB (m mg/kg body weight) treated mice show no signs of liver and renal toxicity in PDX (BCM-4013) model.

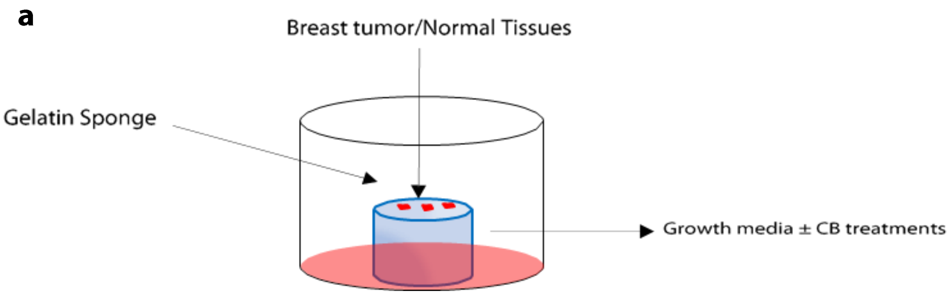

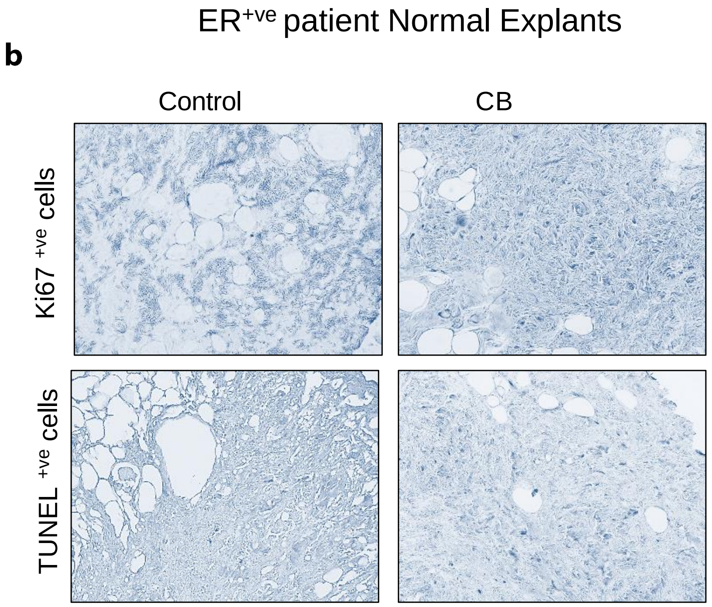

Supplementary Figure 8. Effect of CB on normal breast tissue explants from ER+ve breast cancer patients. (a) Set up for *ex vivo* explants from breast cancer patients. **(b)** Representative images showing immunohistochemical analysis using Ki67 antibody and TUNEL assay in normal breast tissue from ER^{+ve} breast cancer patients treated with either nano-vehicle or nano-CB for 72 hours. Representative photographs are presented at 40x.

Supplementary Figure 9

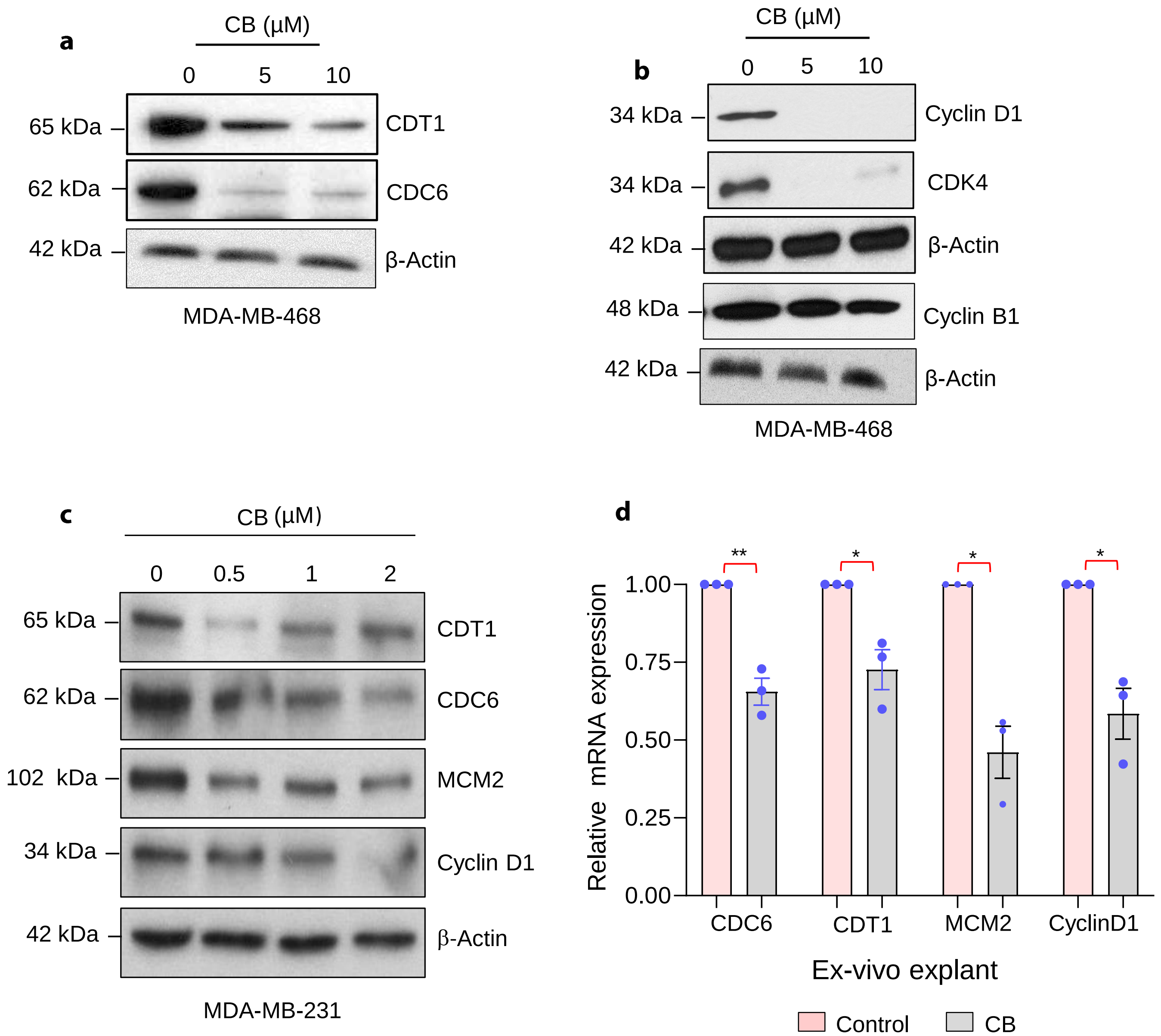

Supplementary Figure 9. CB targets proteins associated with DNA replication and cell cycle progression. (a, b) Western blot analysis of MDA-MB-468 cells treated with vehicle or CB (5 and 10 μM) using antibodies against CDT1 and CDC6 (a); CylD1, CDK4 and Cyc B1 (b). **(c)** Western blot analysis of MDA-MB-231 treated with vehicle (0) or low dose of CB (0.5, 1, 2.0) using antibodies against indicated proteins. **(d)** Real-time qPCR validation of highly altered genes (obtained from microarray analysis) in vehicle control and CB-treated breast cancer patient explants using gene-specific primers. The relative expression of each gene was quantified by measuring Ct values and normalized with GAPDH.

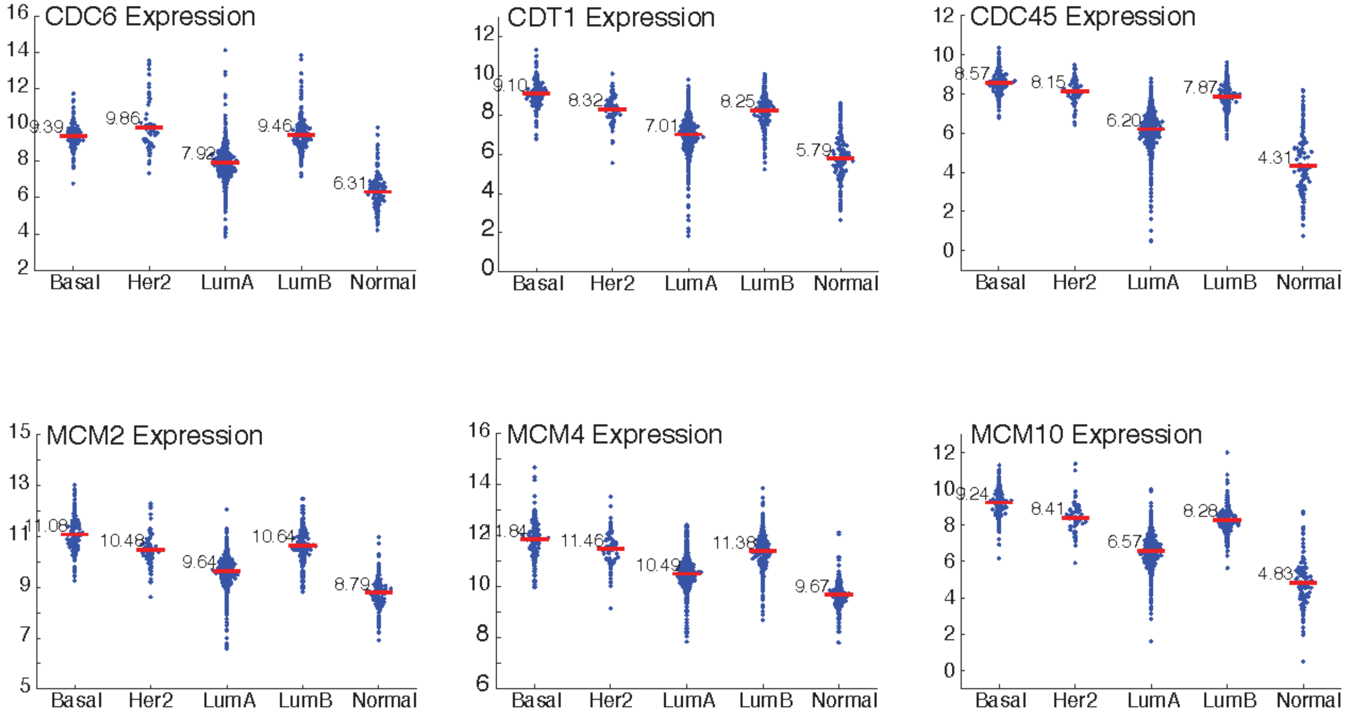

Supplementary Figure 10. Replication licensing genes are significantly altered in all subtypes of breast cancer. TCGA data set show replication licensing associated genes are differentially expressed in different subtype of breast cancer patients.

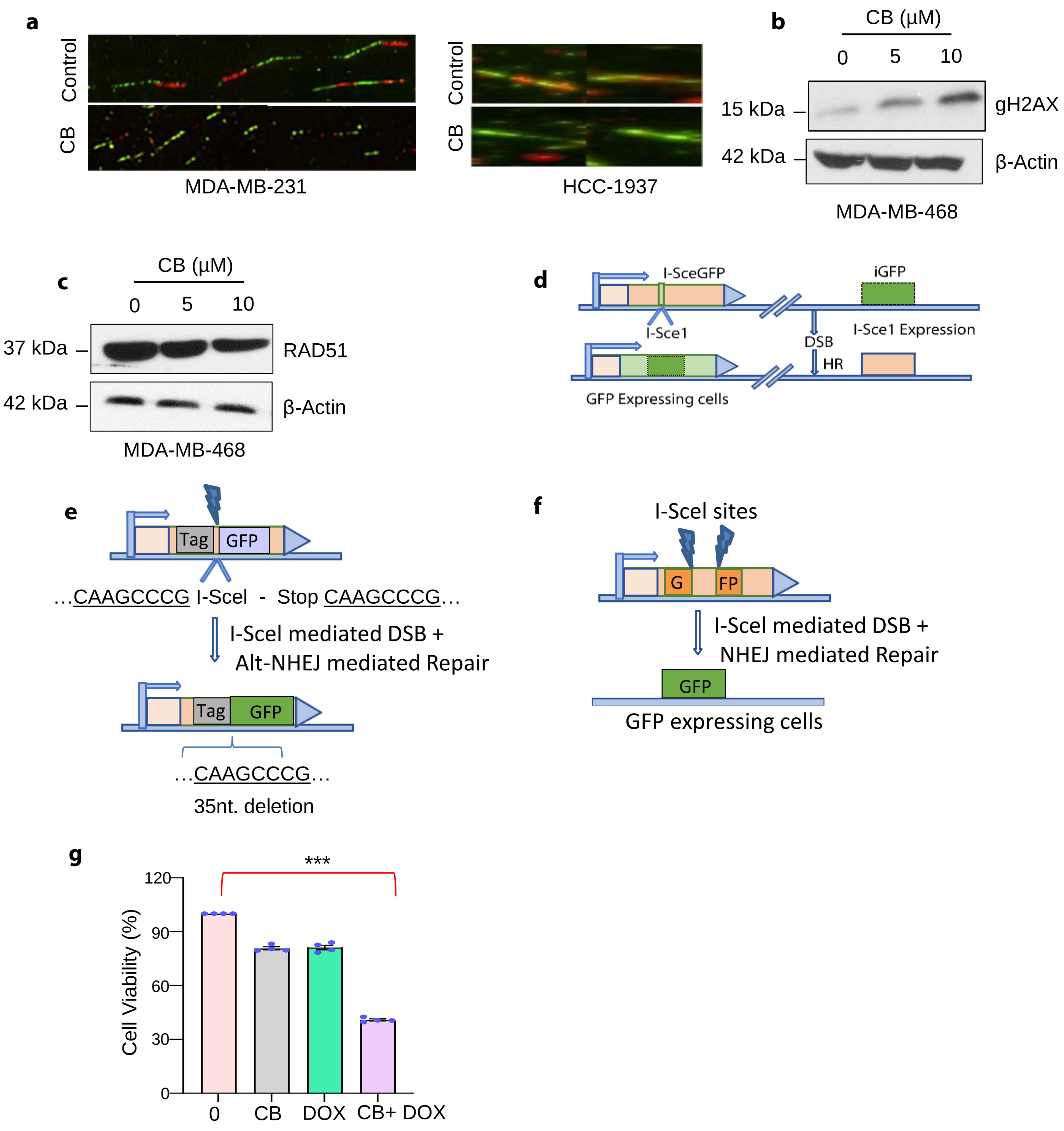

Supplementary Figure 11. CB blocks DNA repair ability of cancer cells improves efficacy of doxorubicin. (a) Representative images of DNA fibers from vehicle and CB-treated MDA-MB-231 and HCC-1937 cells. Red/green fibers show progressing forks, while green fibers show stalled forks and red fibers show new origins. **(b, c)** Western blot analysis of MDA-MB-468 cells treated with vehicle or CB using antibodies against γH2AX (b) RAD51 (c). Membranes were reprobed with β-actin for loading control. **(d)** Schematic of DR-GFP assay to measure homologous recombination-mediated DNA repair. **(e, f)** Schematic of EJ2 and EJ5-GFP assay to measure alt-NHEJ (e) and NHEJ (f)-mediated DNA repair. **(g)** MDA-MB-231 cells are treated with vehicle, CB (200 nM), DOX (5 nM) and CB+DOX combination for 72hrs. Cell viability was assessed using Cell Titer-Glo Luminescent viability assay. ***, $p<0.001$; ****, $p<0.0001$ versus Vehicle group, t test; CB-Carbazole blue, DOX-Doxorubicin.

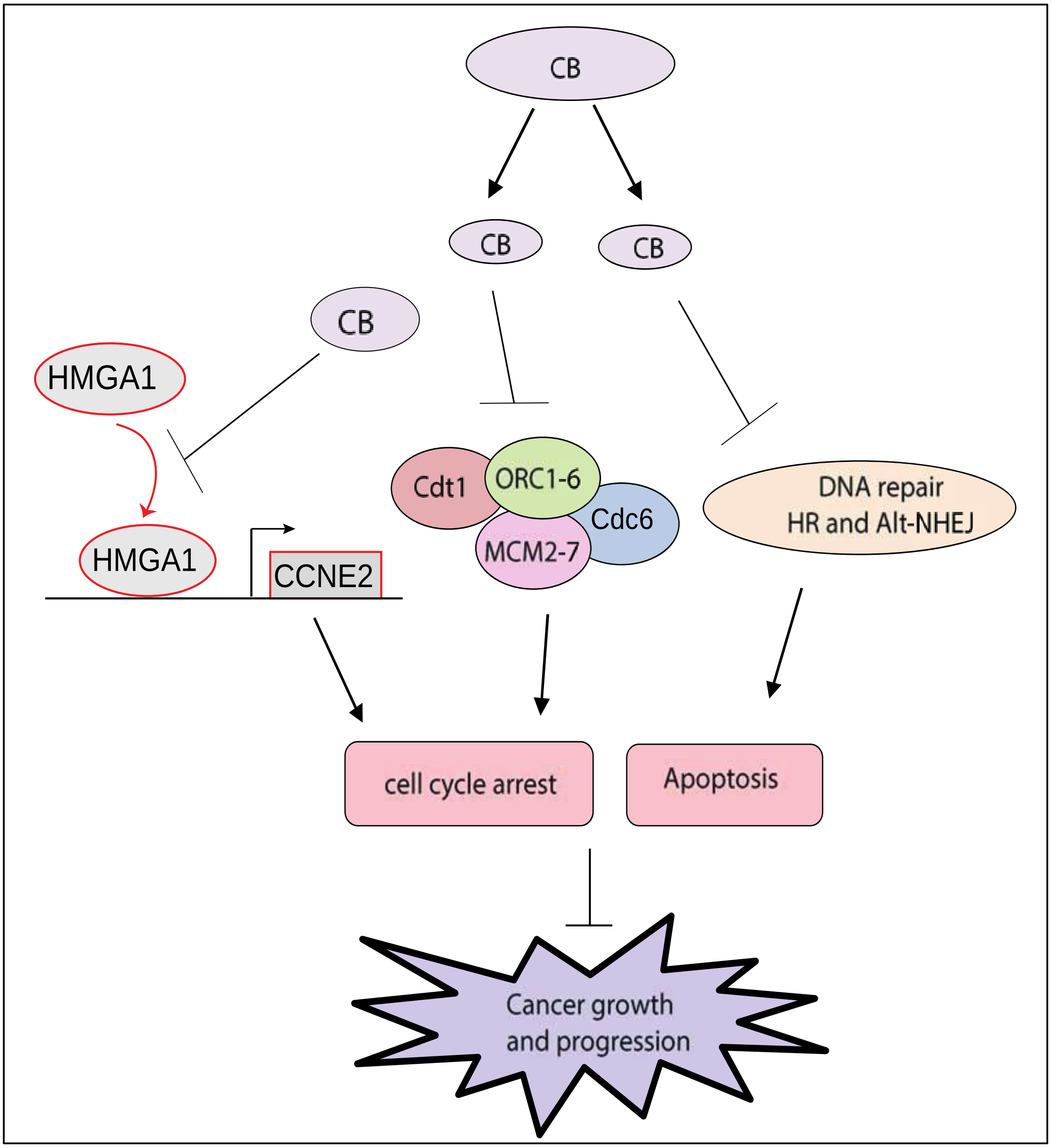

Supplementary Figure 12. CB inhibits breast cancer growth. Model showing CB mode of action. We suggest that CB specifically binds to A-T rich regions of DNA thereby blocking recruitment of AT-rich sequence binding factors such as HMGA1 to target gene promoters and consequently inhibiting target genes expression. This in turn leads to cell cycle and replication arrest and induction of apoptosis in cancer cells.

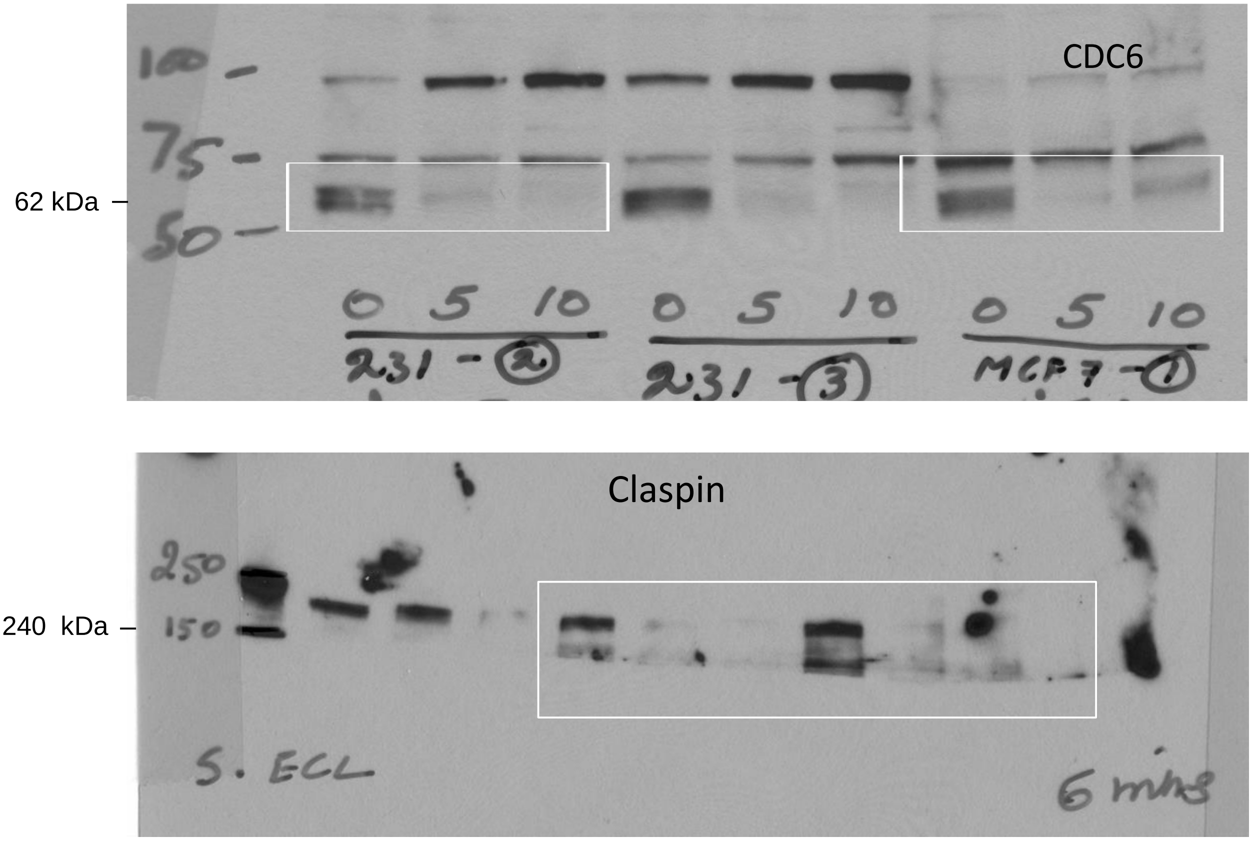

Supplementary Figure 13. CB inhibits CDC6 and Claspin expression. Raw blots showing western blot analysis on vehicle (0) and CB (indicated doses)-treated breast cancer cells using antibodies against indicated proteins. Box with white line indicates results shown in Fig. 5.

Supplementary Figure 14

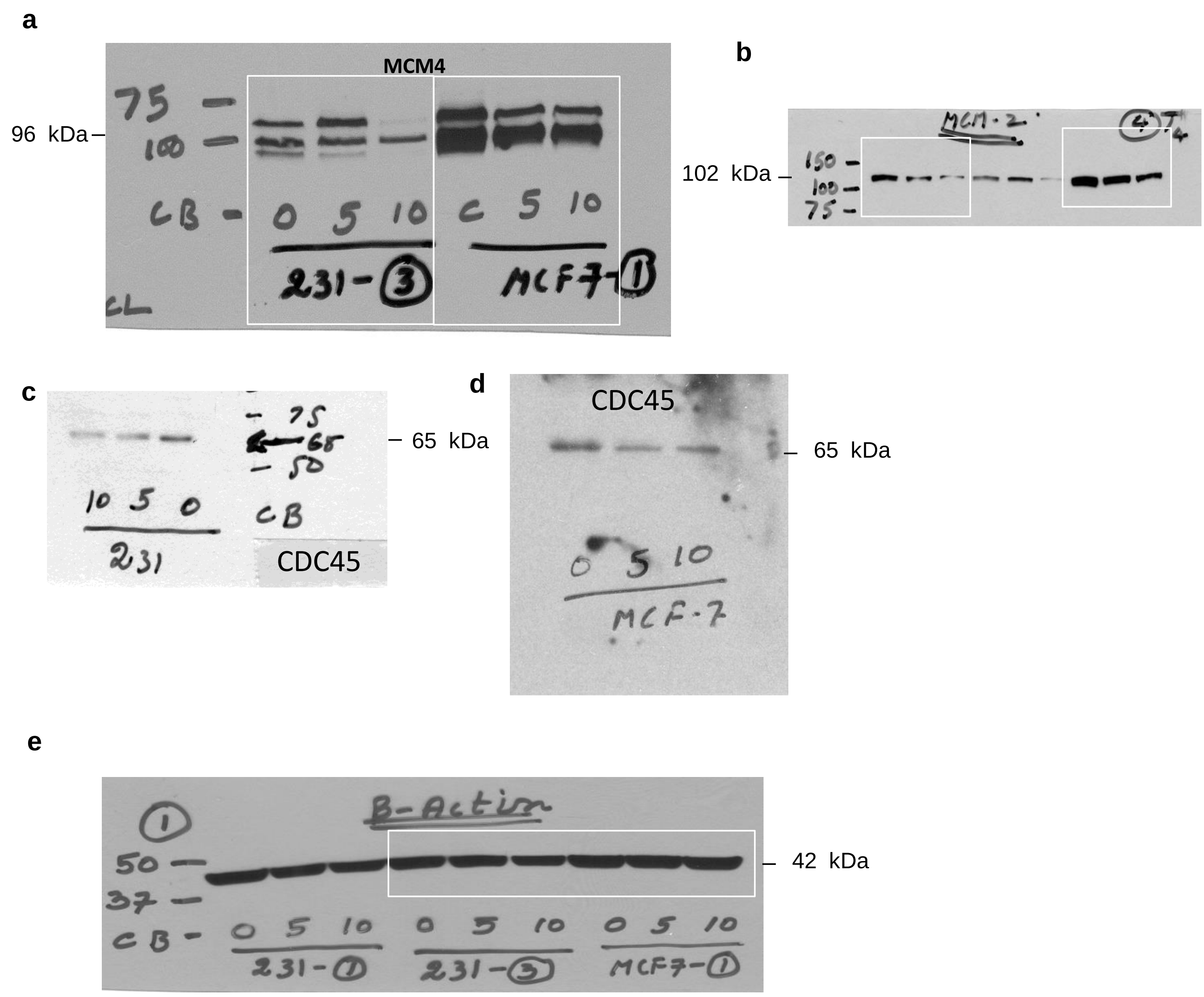

Supplementary Figure 14. CB inhibits target expression. Raw blots showing western blot analysis on vehicle (0) and CB (indicated doses)-treated breast cancer cells using antibodies against indicated proteins. Box with white line indicates results shown in Fig. 5.

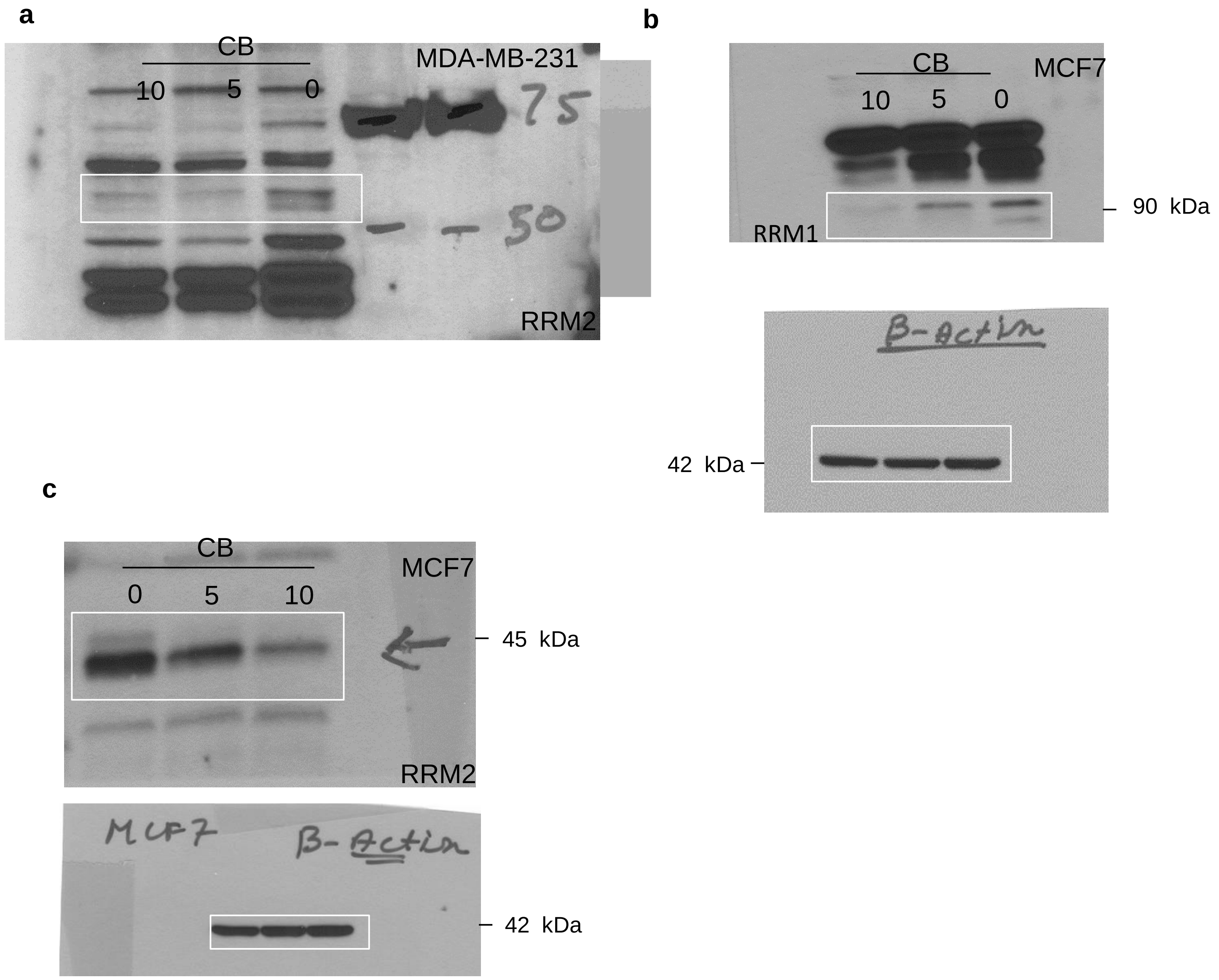

Supplementary Figure 15. CB inhibits target expression. Raw blots showing western blot analysis on vehicle (0) and CB (indicated doses)-treated breast cancer cells using antibodies against indicated proteins. Box with white line indicates results shown in Fig. 5.

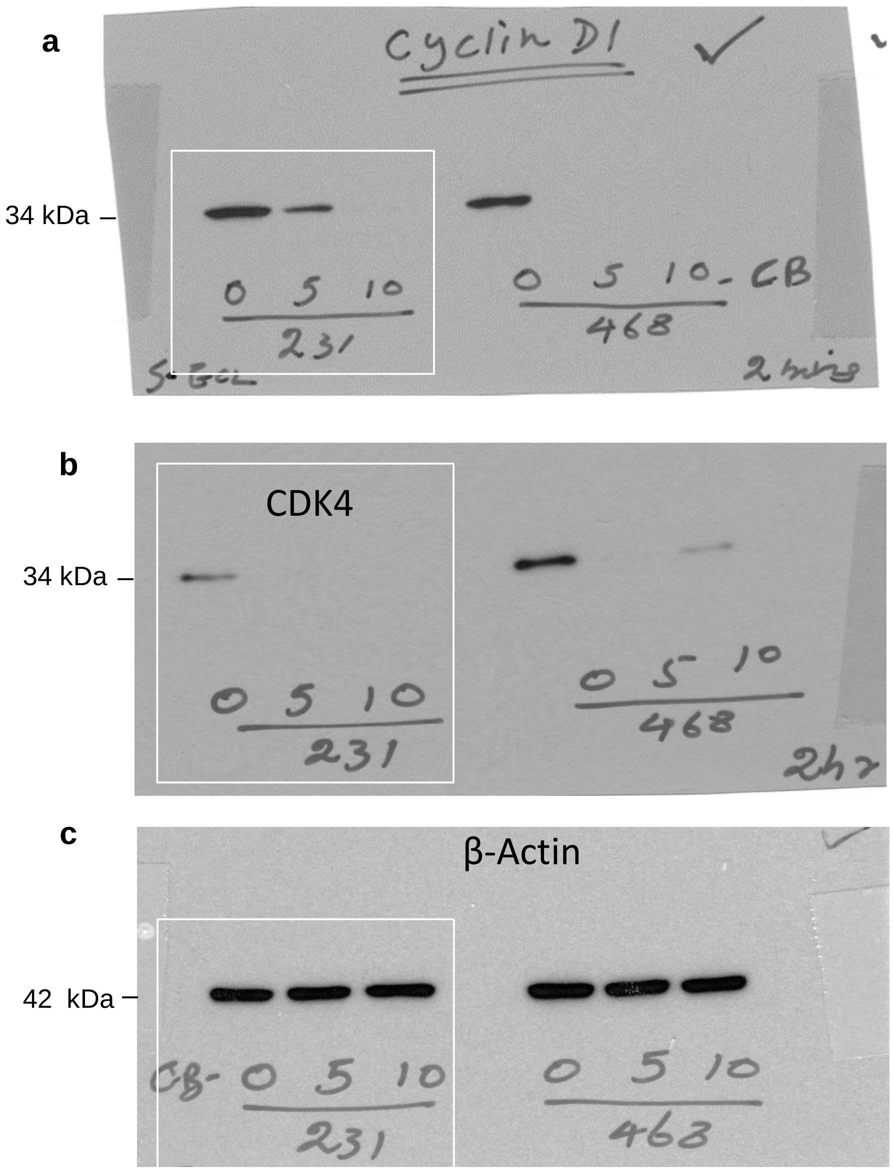

Supplementary Figure 16. CB inhibits target expression. Raw blots showing western blot analysis on vehicle (0) and CB (indicated doses)-treated breast cancer cells using antibodies against indicated proteins. Box with white line indicates results shown in Fig. 6.

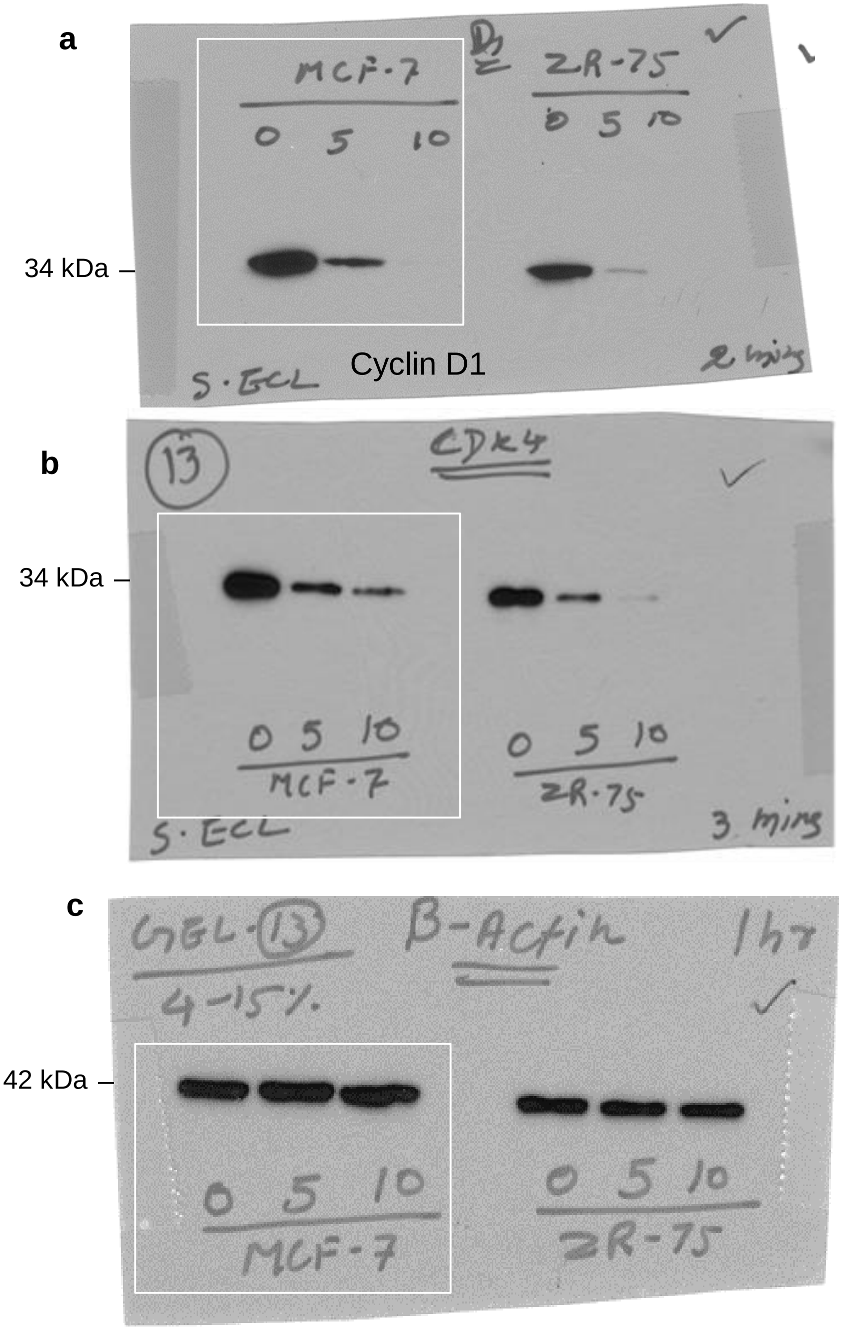

Supplementary Figure 17. CB inhibits target expression. Raw blots showing western blot analysis on vehicle (0) and CB (indicated doses)-treated breast cancer cells using antibodies against indicated proteins. Box with white line indicates results shown in Fig. 6.

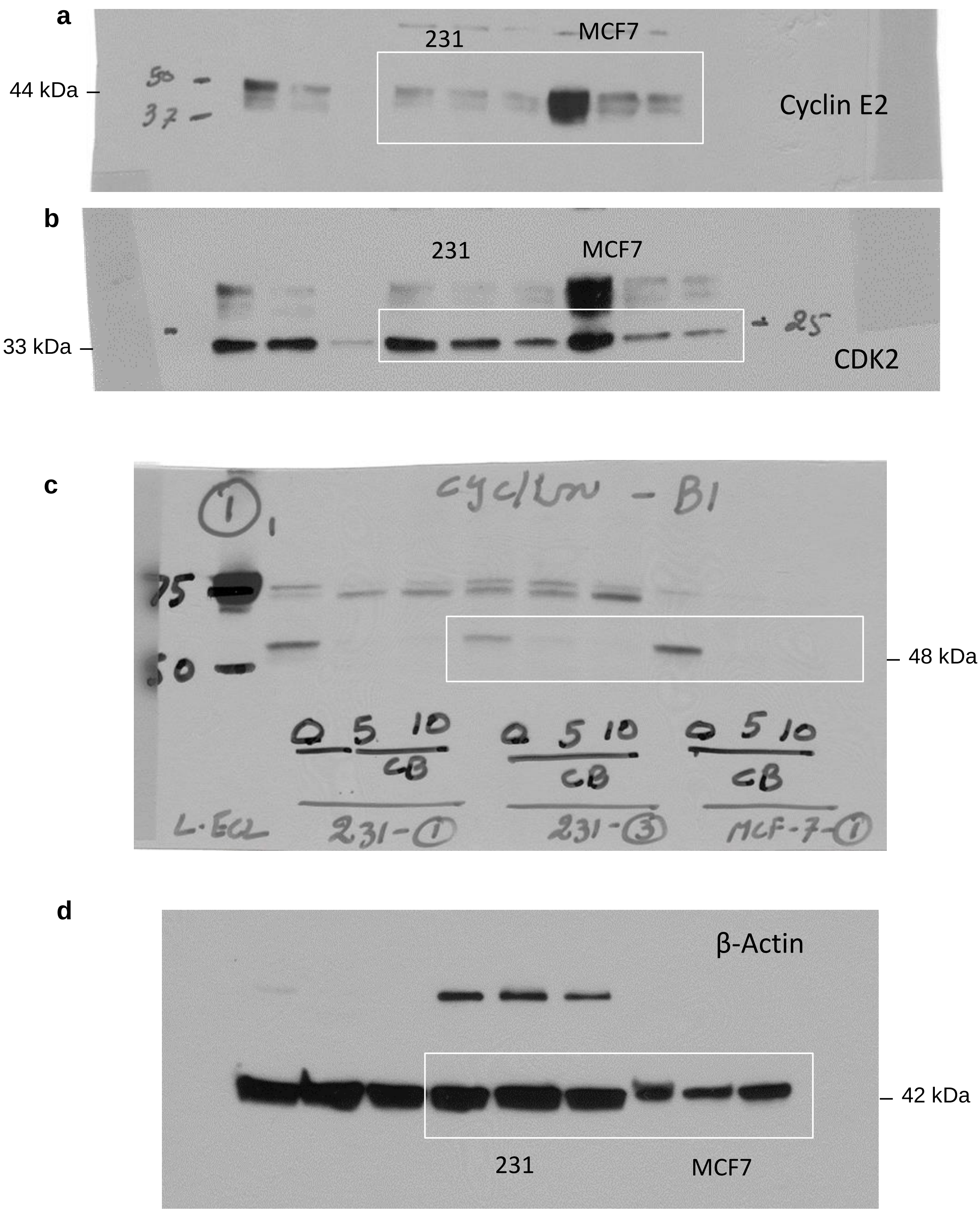

Supplementary Figure 18. CB inhibits target expression. Raw blots showing western blot analysis on vehicle (0) and CB (indicated doses)-treated breast cancer cells using antibodies against indicated proteins. Box with white line indicates results shown in Fig. 6.

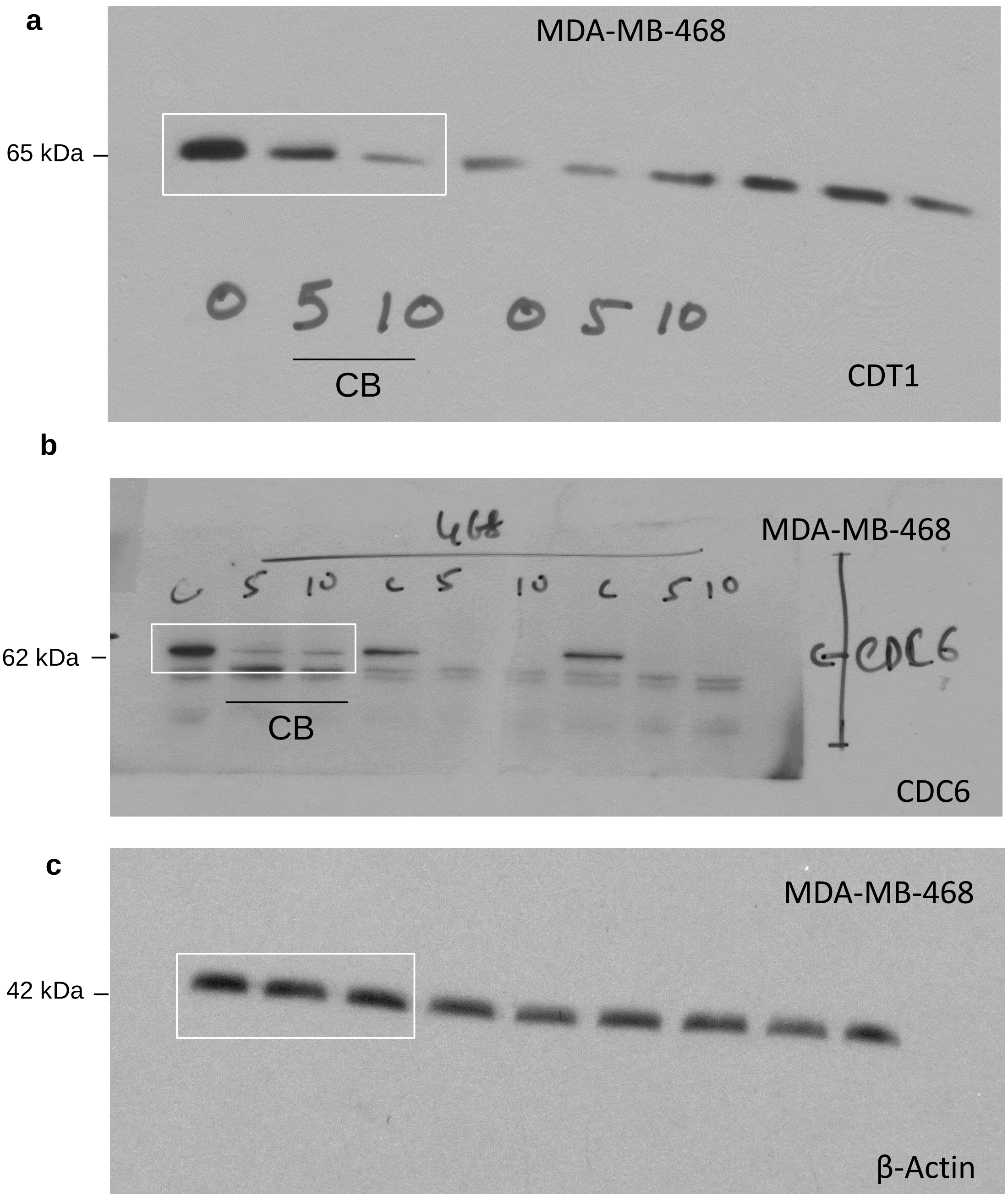

Supplementary Figure 19. CB inhibits the expression of cell cycle proteins. Raw blots showing western blot analysis on vehicle (0) and CB (indicated doses)-treated breast cancer cells using antibodies against indicated proteins. Box with white line indicates results shown in Supplementary Figure 9.

Supplementary Figure 20

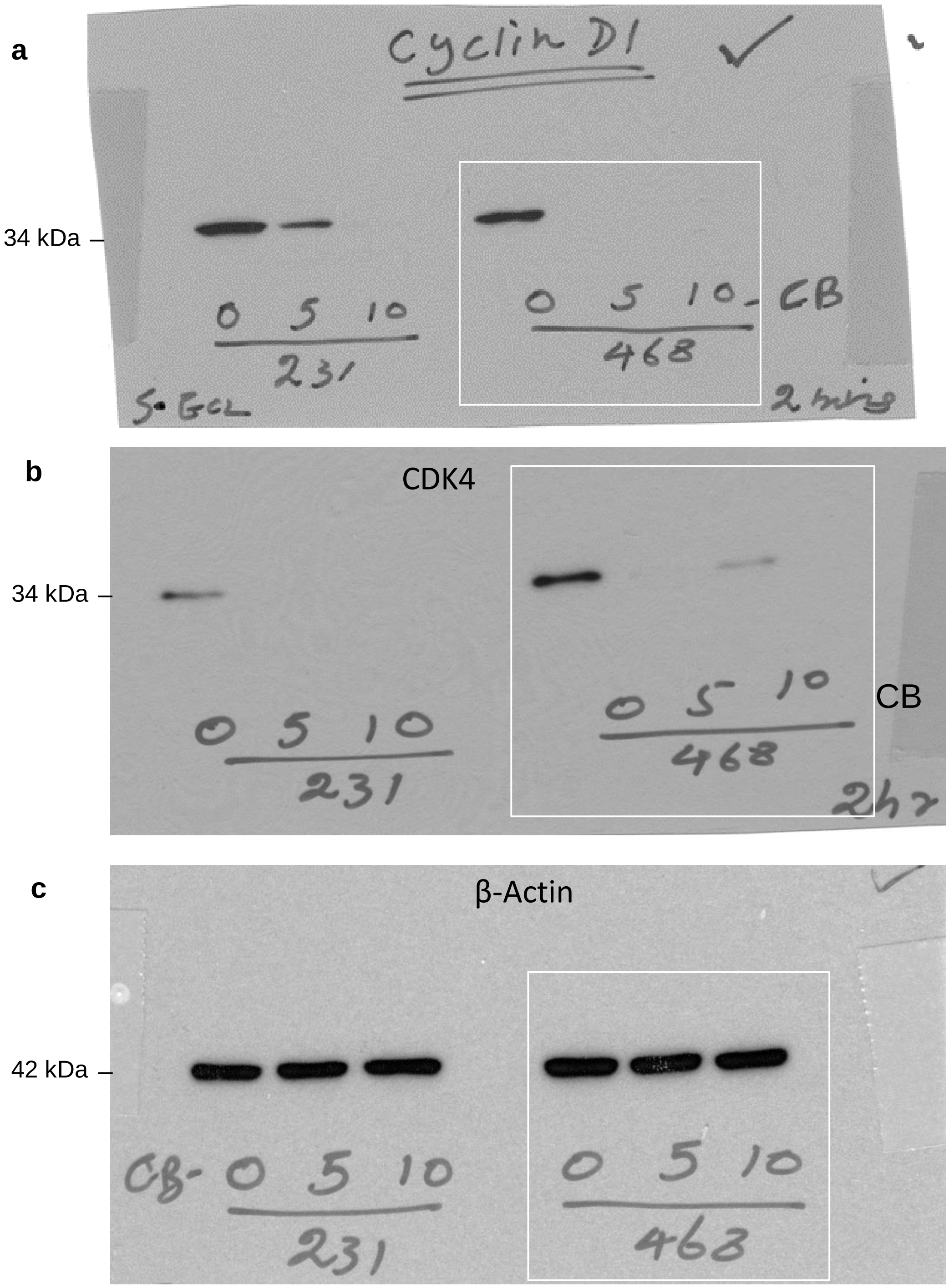

Supplementary Figure 20. CB inhibits the expression of cell cycle proteins. Raw blots showing western blot analysis on vehicle (0) and CB (indicated doses)-treated breast cancer cells using antibodies against indicated proteins. Box with white line indicates results shown in Supplementary Figure 9.

a

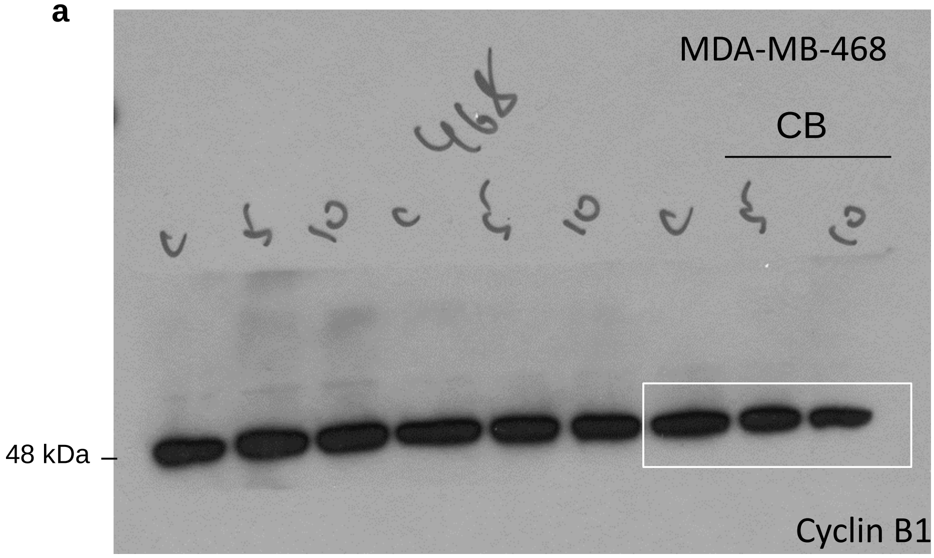

b

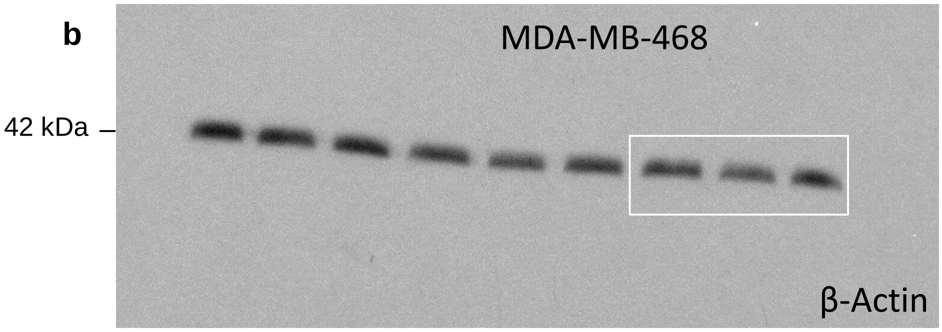

Supplementary Figure 21. CB inhibits the expression of cell cycle protein cyclin B1. Raw blots showing western blot analysis on vehicle (0) and CB (indicated doses)-treated breast cancer cells using antibodies against indicated proteins. Box with white line indicates results shown in Supplementary Figure 9.

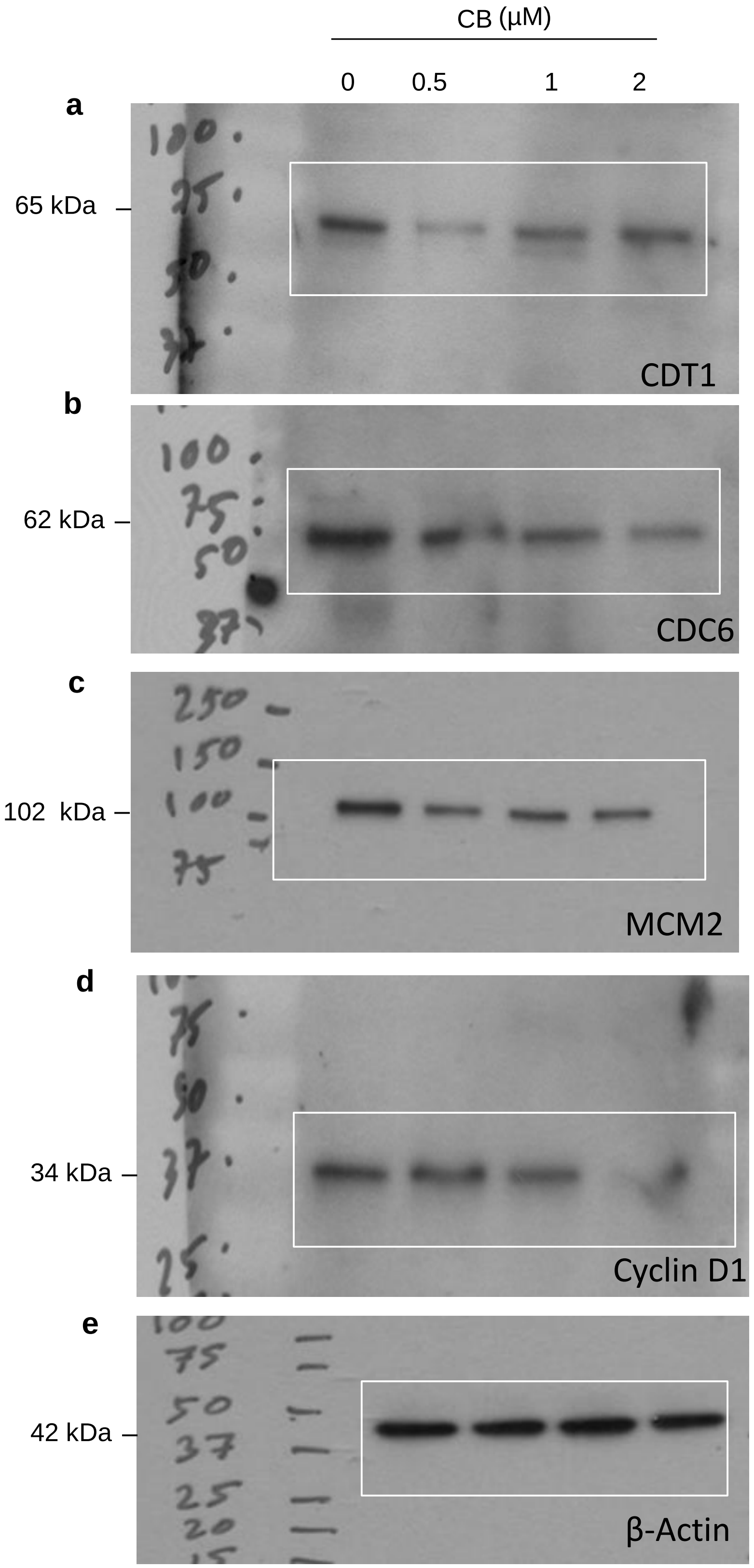

Supplementary Figure 22. CB inhibits the expression of cell cycle and replication associated proteins. Raw blots showing western blot analysis on vehicle (0) and CB (indicated doses)-treated MDA-MB-231 breast cancer cells using antibodies against indicated proteins. Box with white line indicates results shown in Supplementary Figure 9.

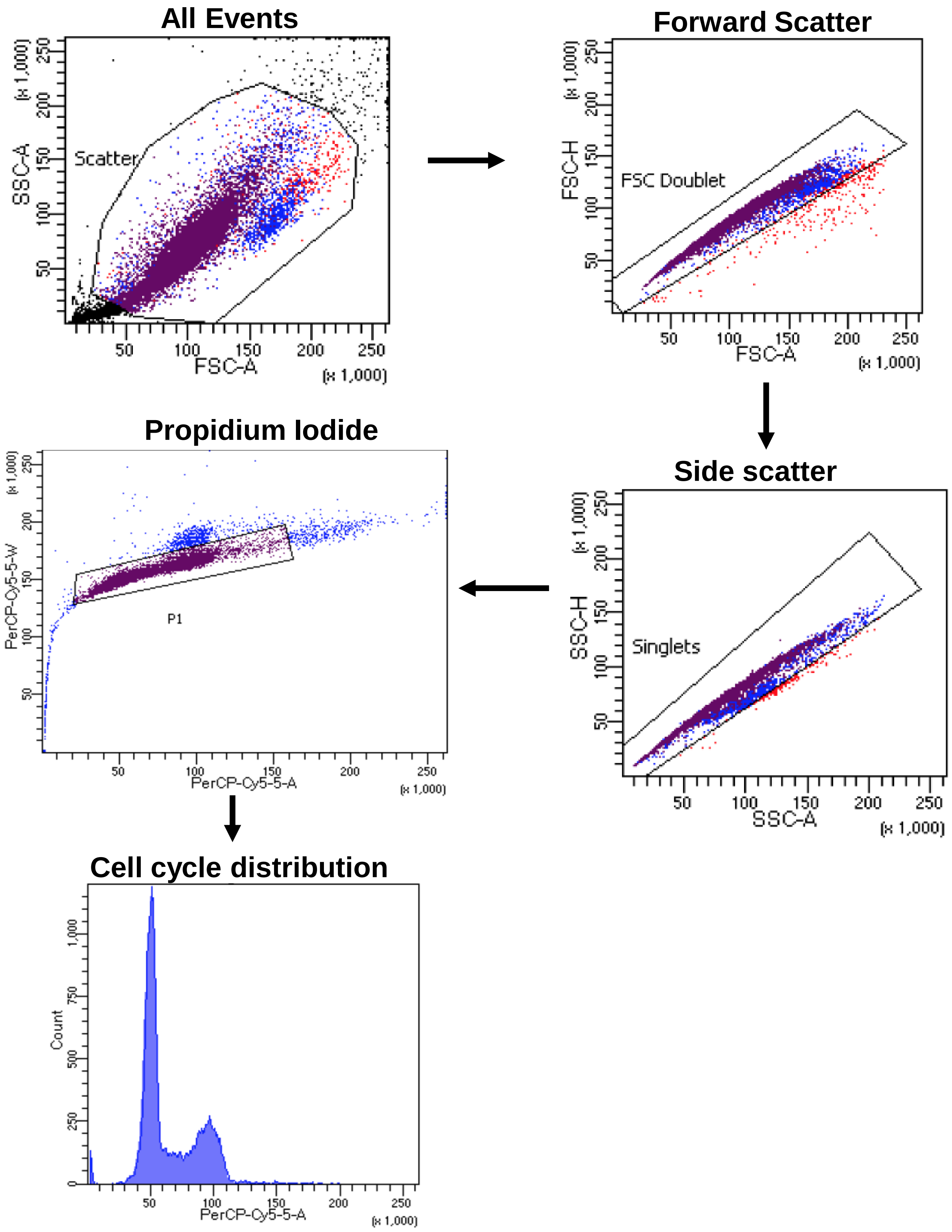

Supplementary Figure 23. Gating strategy for Propidium Iodide-based Cell cycle analysis in Vehicle and CB treated breast cancer cells (as shown in Fig 6A).

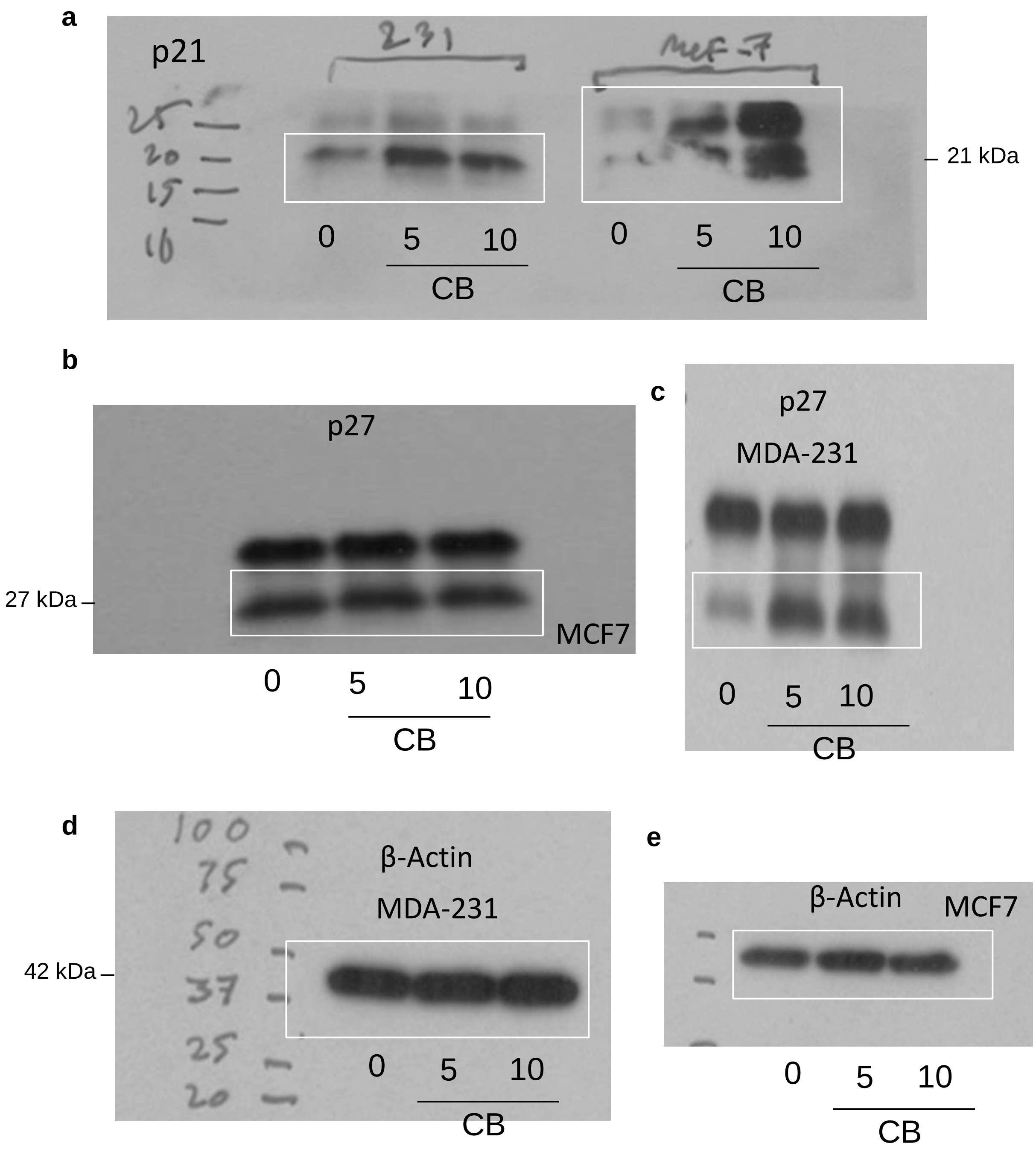

Supplementary Figure 24. CB induces the expression of p21 and p27 in breast cancer cells. Raw blots showing western blot analysis on vehicle (0) and CB (indicated doses)-treated breast cancer cells using antibodies against indicated proteins. Box with white line indicates results shown in Fig. 6.

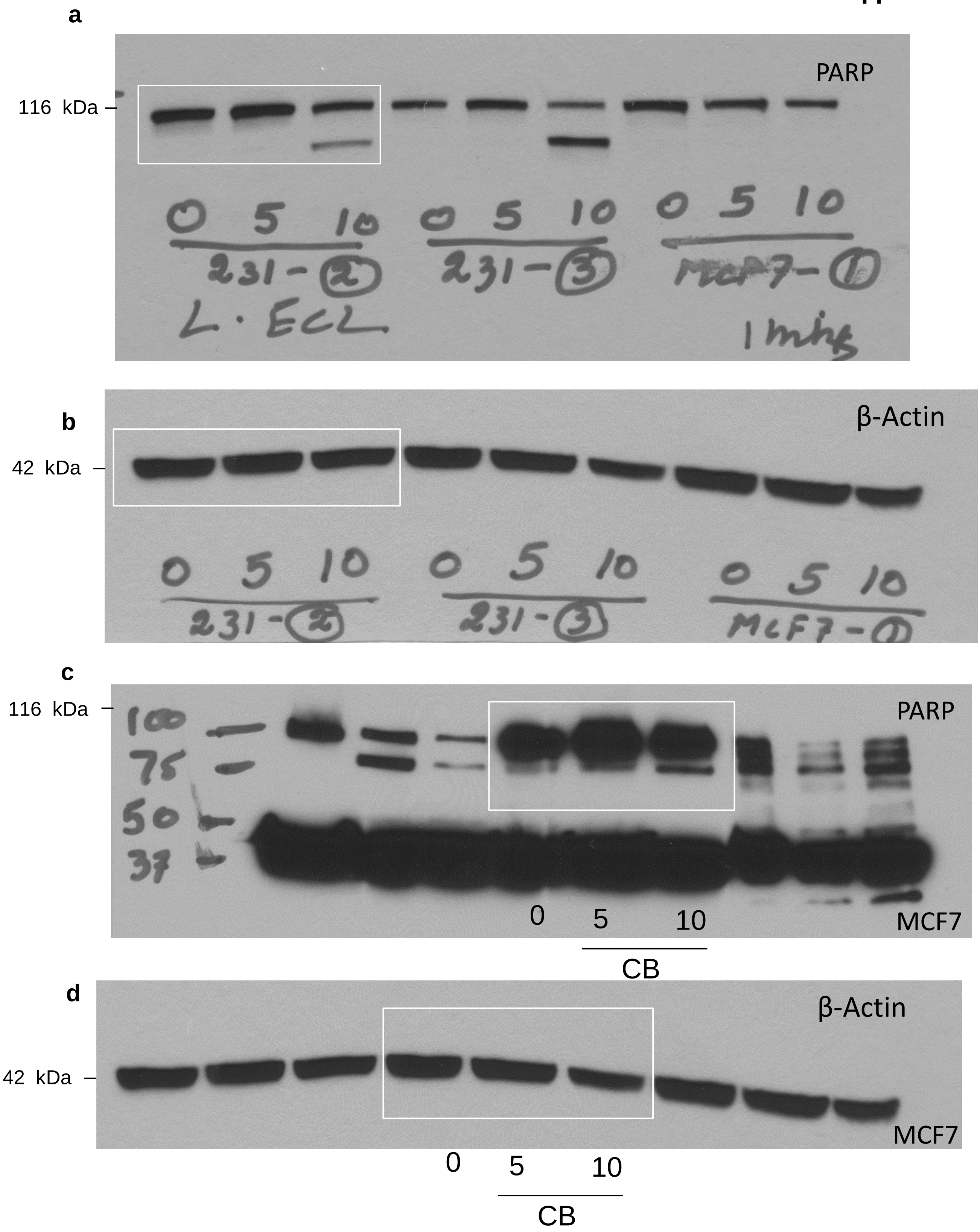

Supplementary Figure 25. CB induces PARP cleavage in breast cancer cells. Raw blots showing western blot analysis on vehicle (0) and CB (indicated doses)-treated breast cancer cells using antibodies against indicated proteins. Box with white line indicates results shown in Fig. 6.

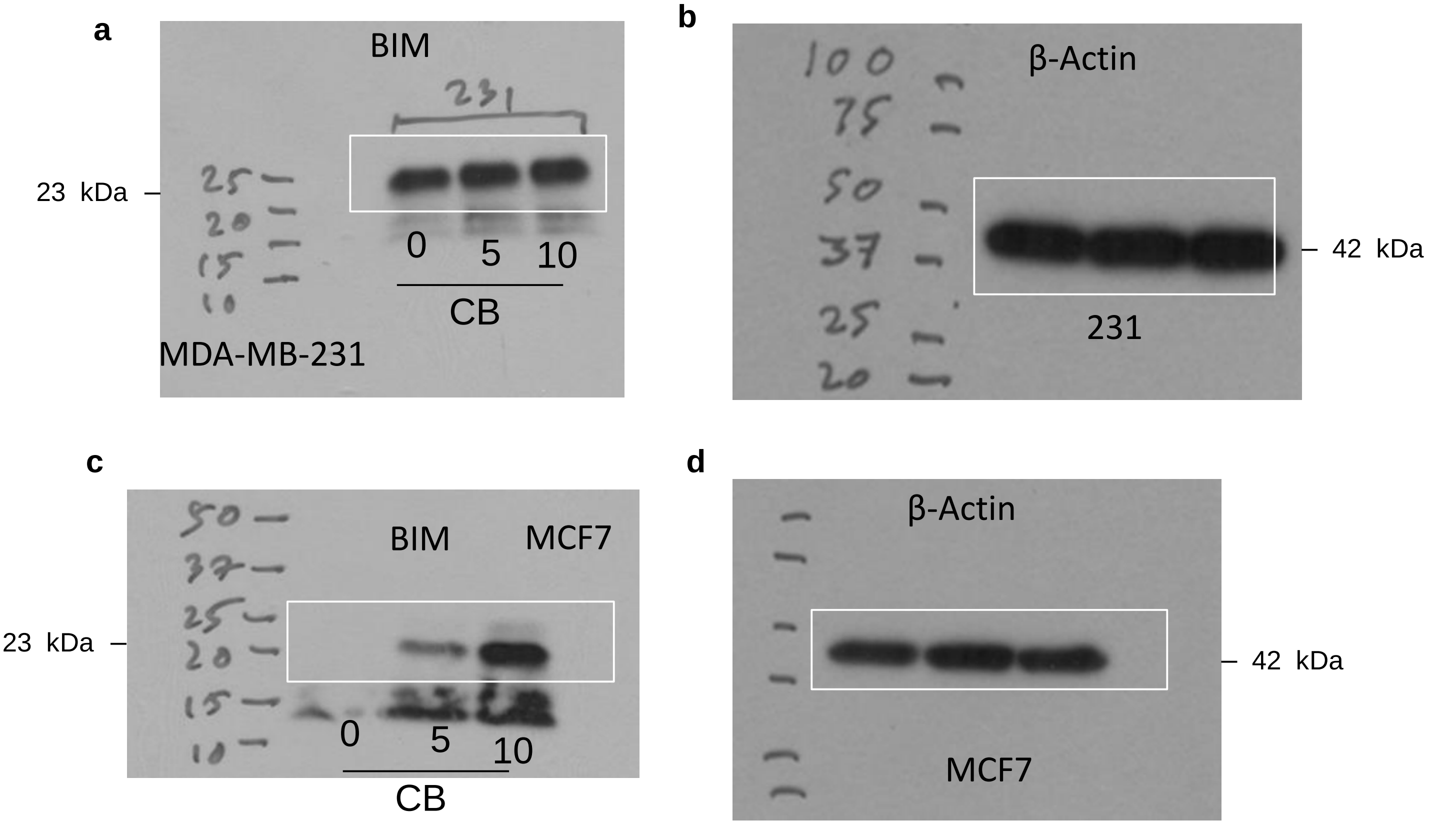

Supplementary Figure 26. CB induces the expression of apoptotic protein BIM. Raw blots showing western blot analysis on vehicle (0) and CB (indicated doses)-treated breast cancer cells using antibodies against indicated proteins. Box with white line indicates results shown in Fig. 6.

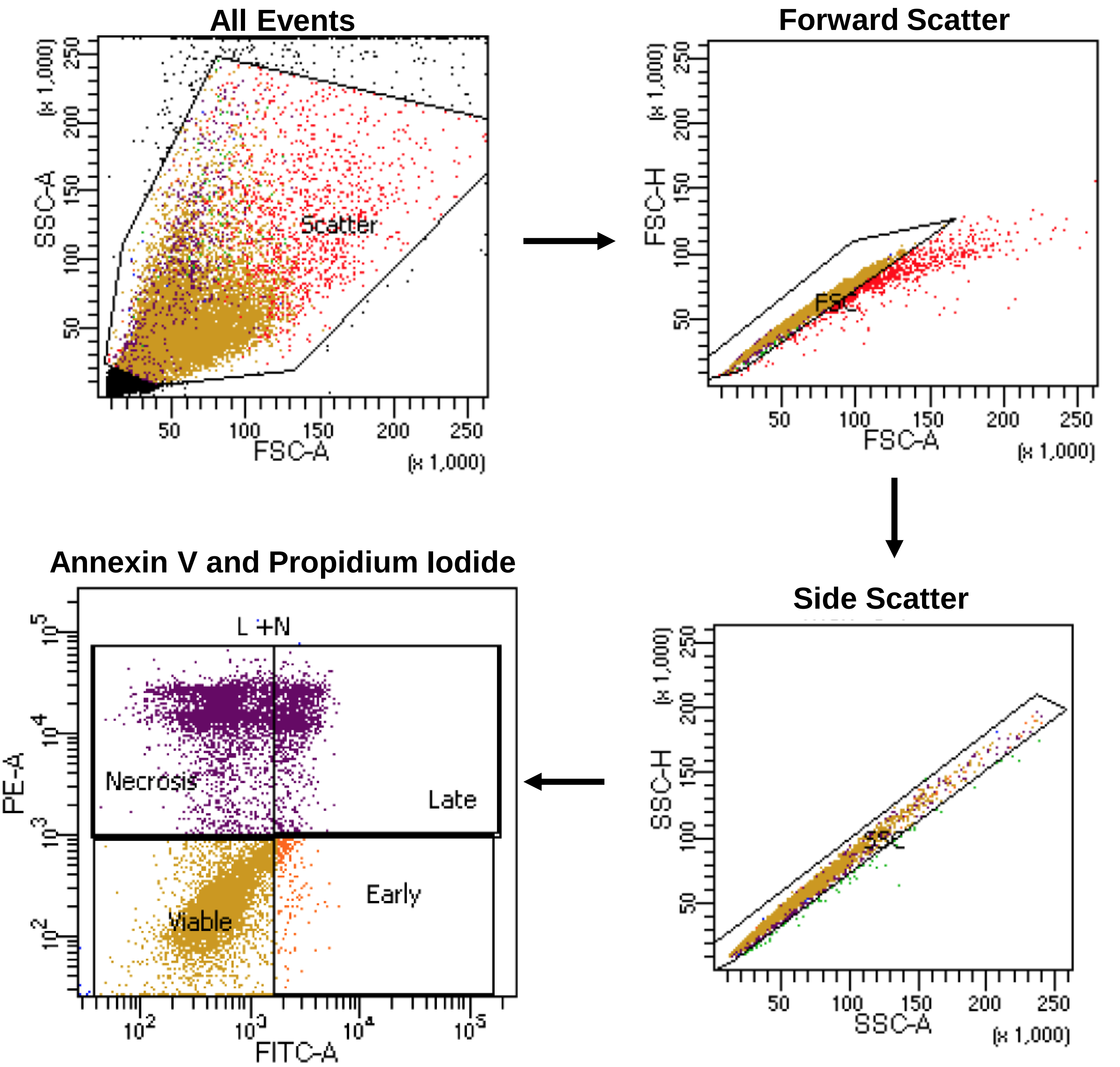

Supplementary Figure 27. Gating strategy for Annexin V-FITC and Propidium Iodide-based apoptosis assay in Vehicle and CB treated breast cancer cells (as shown in Fig 6e).

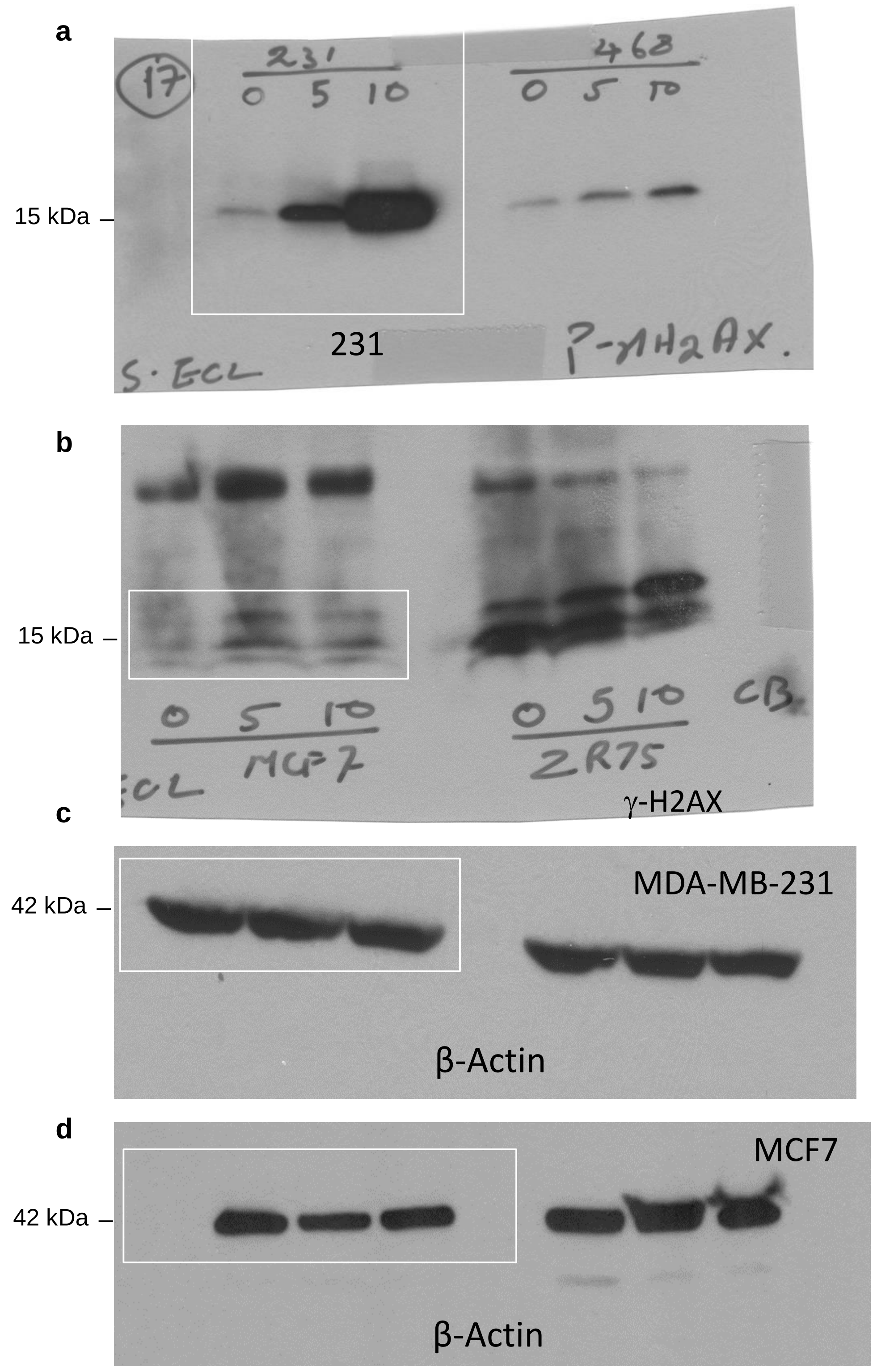

Supplementary Figure 28. CB induces the expression of DNA damage response protein γ-H2AX. Raw blots showing western blot analysis on vehicle (0) and CB (indicated doses)-treated breast cancer cells using antibodies against indicated proteins. Box with white line indicates results shown in Fig. 7.

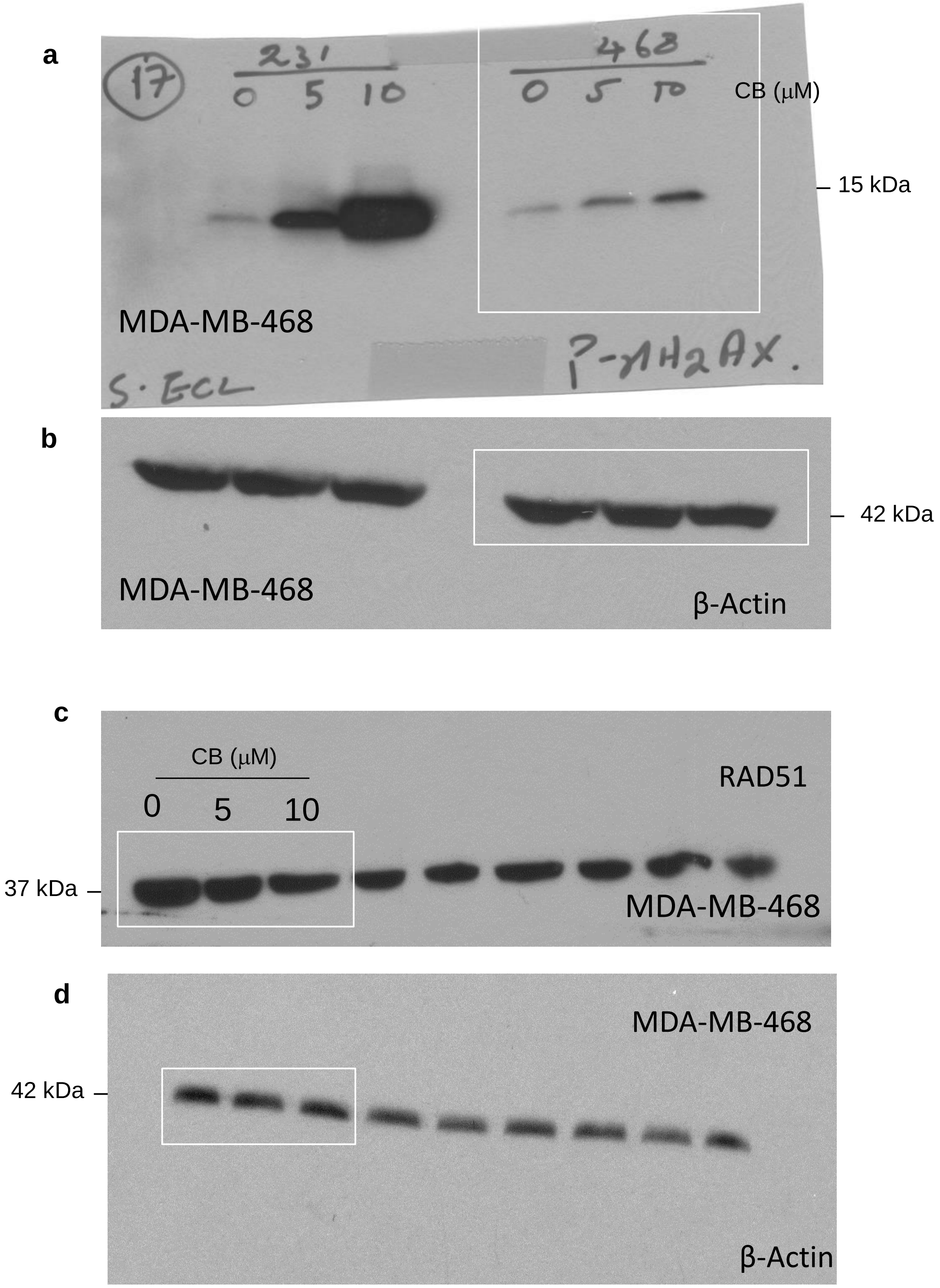

Supplementary Figure 29. CB induces the expression of g-H2AX (a) and inhibits the expression of RAD51 (c). Raw blots showing western blot analysis on vehicle (0) and CB (indicated doses)-treated MDA-MB-468 breast cancer cells using antibodies against indicated proteins. Box with white line indicates results shown in Supplementary Figure 11.

Supplementary Figure 30

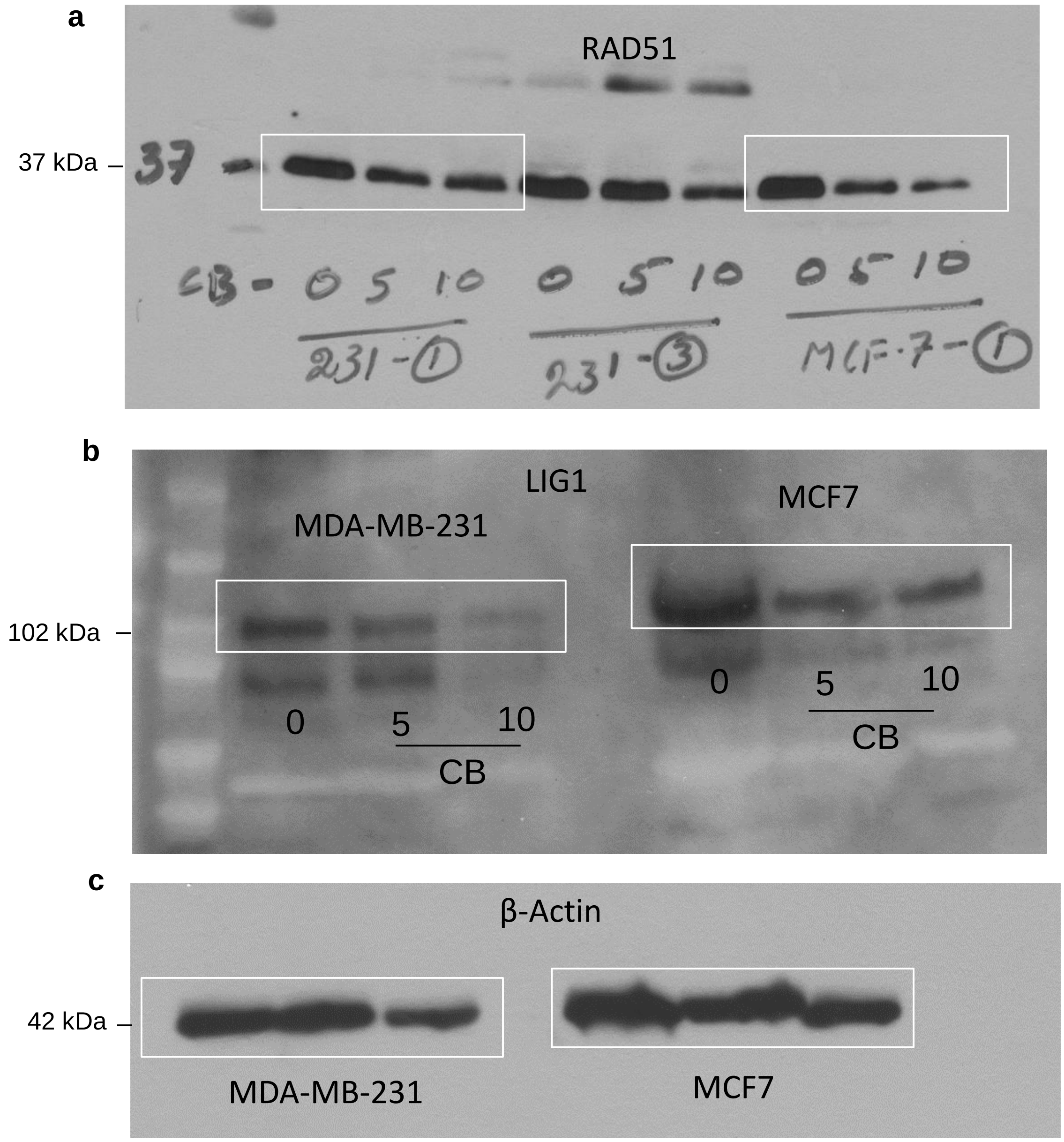

Supplementary Figure 30. CB inhibits the expression of DNA repair proteins RAD51 and LIG1**.** Raw blots showing western blot analysis on vehicle (0) and CB (indicated doses)-treated breast cancer cells using antibodies against indicated proteins. Box with white line indicates results shown in Fig. 7.

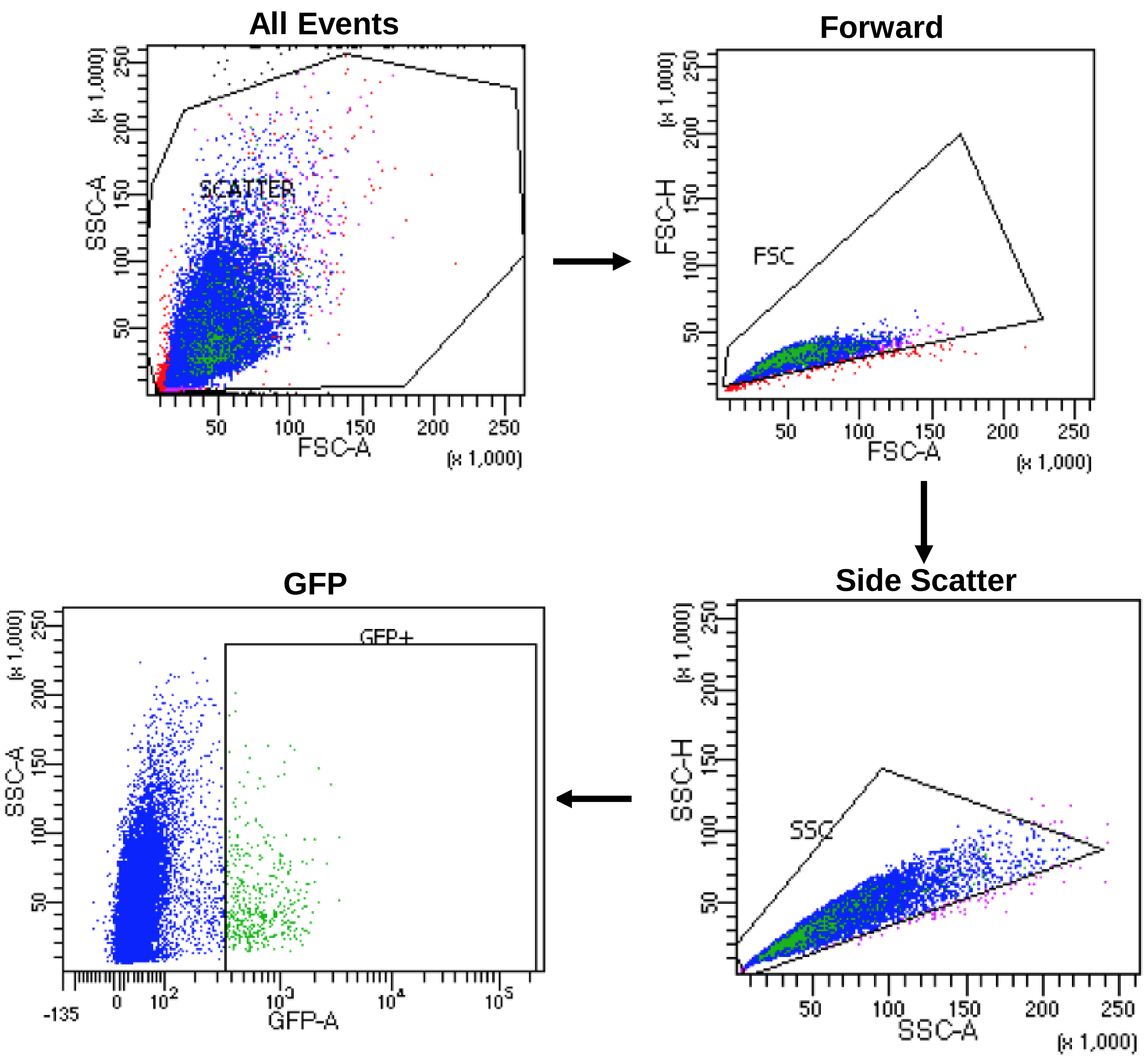

Supplementary Figure 31. Gating strategy for Isce-I based DNA repair assays in Vehicle and CB treated cells (as shown in Fig 7h, 7k and 8b).
