# Supplementary material for: Targeting aberrant replication and DNA repair events for treating breast cancers
Source: Commun Biol. 2022 May 24;5:493. doi: 10.1038/s42003-022-03413-w (PMC9130234; doi:10.1038/s42003-022-03413-w)
Supplement: Supplementary file 2 — Description of Additional Supplementary Files [file 42003_2022_3413_MOESM2_ESM.pdf]

Description of Additional Supplementary Files

File Name: Supplementary Data 1
Description: The source data for graphs and tables shown in the manuscript.

File Name: Supplementary Data 2
Description: Differentially expressed genes derived from comparing CB treated MDA-MB-231 cells versus vehicle control. The source data underlying Figure 5
