# Supplementary material for: Targeting aberrant replication and DNA repair events for treating breast cancers
Source: Commun Biol. 2022 May 24;5:493. doi: 10.1038/s42003-022-03413-w (PMC9130234; doi:10.1038/s42003-022-03413-w)
Supplement: Supplementary file 5 — Reporting Summary [file 42003_2022_3413_MOESM5_ESM.pdf]

nature research

Corresponding author(s): Dr. Manjeet Rao
Last updated by author(s): Mar 7, 2022

Reporting Summary

Nature Research wishes to improve the reproducibility of the work that we publish. This form provides structure for consistency and transparency in reporting. For further information on Nature Research policies, see our Editorial Policies and the Editorial Policy Checklist.

Statistics

For all statistical analyses, confirm that the following items are present in the figure legend, table legend, main text, or Methods section.

n/a	Confirmed	
☐	☒	The exact sample size (n) for each experimental group/condition, given as a discrete number and unit of measurement
☐	☒	A statement on whether measurements were taken from distinct samples or whether the same sample was measured repeatedly
☐	☒	The statistical test(s) used AND whether they are one- or two-sided<br>*Only common tests should be described solely by name; describe more complex techniques in the Methods section.*
☒	☐	A description of all covariates tested
☒	☐	A description of any assumptions or corrections, such as tests of normality and adjustment for multiple comparisons
☐	☒	A full description of the statistical parameters including central tendency (e.g. means) or other basic estimates (e.g. regression coefficient) AND variation (e.g. standard deviation) or associated estimates of uncertainty (e.g. confidence intervals)
☒	☐	For null hypothesis testing, the test statistic (e.g. F, t, r) with confidence intervals, effect sizes, degrees of freedom and P value noted<br>*Give P values as exact values whenever suitable.*
☒	☐	For Bayesian analysis, information on the choice of priors and Markov chain Monte Carlo settings
☒	☐	For hierarchical and complex designs, identification of the appropriate level for tests and full reporting of outcomes
☐	☒	Estimates of effect sizes (e.g. Cohen's d, Pearson's r), indicating how they were calculated

Our web collection on statistics for biologists contains articles on many of the points above.

Software and code

Policy information about availability of computer code

Data collection	Not applicable
Data analysis	Not applicable

For manuscripts utilizing custom algorithms or software that are central to the research but not yet described in published literature, software must be made available to editors and reviewers. We strongly encourage code deposition in a community repository (e.g. GitHub). See the Nature Research guidelines for submitting code & software for further information.

Data

Policy information about availability of data

All manuscripts must include a data availability statement. This statement should provide the following information, where applicable:

- Accession codes, unique identifiers, or web links for publicly available datasets
- A list of figures that have associated raw data
- A description of any restrictions on data availability

All raw data associated with the manuscript will be available one year after publication of the manuscript. Fig. 5A and Table 2 are derived from microarray analysis. Raw data for microarray submitted to GEO ((GSE161911) [NCBI tracking system #21459658].

Field-specific reporting

Please select the one below that is the best fit for your research. If you are not sure, read the appropriate sections before making your selection.

☒ Life sciences ☐ Behavioural & social sciences ☐ Ecological, evolutionary & environmental sciences

For a reference copy of the document with all sections, see nature.com/documents/nr-reporting-summary-flat.pdf

Life sciences study design

All studies must disclose on these points even when the disclosure is negative.

Sample size	The minimum sample size of 7 mice for each experimental group was determined using Time-Averaged Difference Power Analysis to detect a 50 percent reduction in treated tumor cell volume with power >0.80 %, autocorrelation=0.5, a compound symmetric within-subject covariance matrix, and α=0.05(1-sided).<br>The minimum sample size of 6 mice for each experimental group was determined using Time-Averaged Difference Power Analysis to detect a 50 percent reduction in treated tumor cell volume with power >0.80%, with 8-repeated measures, autocorrelation=0.5, a compound symmetric within-subject covariance matrix when the standard deviation is 0.4, and α=0.05(1-sided)
Data exclusions	No data were excluded
Replication	All experiments are repeated independently at least three times (biological replicates).
Randomization	Mice were randomly divided into control and treatment groups. Group 1 served as controls and received vehicle (DMSO). Groups 2 and 3 received CB conjugated with liposome nanoparticles (1.5 and 3mg/kg/body weight in 20% intralipid)
Blinding	Investigators were blinded to all cell line and animal experiments.

Reporting for specific materials, systems and methods

We require information from authors about some types of materials, experimental systems and methods used in many studies. Here, indicate whether each material, system or method listed is relevant to your study. If you are not sure if a list item applies to your research, read the appropriate section before selecting a response.

Materials & experimental systems

n/a	Involved in the study	
☐	☒	Antibodies
☐	☒	Eukaryotic cell lines
☒	☐	Palaeontology and archaeology
☐	☒	Animals and other organisms
☒	☐	Human research participants
☒	☐	Clinical data
☒	☐	Dual use research of concern

Methods

n/a	Involved in the study	
☒	☐	ChIP-seq
☐	☒	Flow cytometry
☒	☐	MRI-based neuroimaging

Antibodies

Antibodies used	Mentioned in the Supplementary Table 1
Validation	Antibodies that were either used in previous publications or reported to be validated on vendors website were used for this study.

Eukaryotic cell lines

Policy information about cell lines

Cell line source(s)	ATCC
Authentication	Cell lines were purchased directly from ATCC
Mycoplasma contamination	Nil
Commonly misidentified lines (See ICLAC register)	Nil

Animals and other organisms

Policy information about studies involving animals; ARRIVE guidelines recommended for reporting animal research

Laboratory animals	Mentioned in the Methods section of Manuscript
Wild animals	N/A
Field-collected samples	Mentioned in the Methods section of Manuscript
Ethics oversight	Mentioned in the Methods section of Manuscript

Note that full information on the approval of the study protocol must also be provided in the manuscript.

Flow Cytometry

Plots

Confirm that:

☒ The axis labels state the marker and fluorochrome used (e.g. CD4-FITC).

☒ The axis scales are clearly visible. Include numbers along axes only for bottom left plot of group (a 'group' is an analysis of identical markers).

☒ All plots are contour plots with outliers or pseudocolor plots.

☒ A numerical value for number of cells or percentage (with statistics) is provided.

Methodology

Sample preparation	Mentioned in the Methods section of Manuscript
Instrument	Mentioned in the Methods section of Manuscript
Software	Mentioned in the Methods section of Manuscript
Cell population abundance	Mentioned in the Methods section of Manuscript
Gating strategy	Gating Strategy for cell cycle analysis (Fig. 6a): All Events--> forward scatter --> side scatter--> PerCP-Cy-5.5-Area vs PerCP-Cy-5.5-Width<br><br>Gating Strategy for Annexin V analysis (Fig. 6e):All Events--> forward scatter --> side scatter--> PE-A and FITC-A<br><br>Gating strategy for DNA repair assays (Figs. 7h, 7k and 8b): All Events--> forward scatter --> side scatter--> GFP-A

☒ Tick this box to confirm that a figure exemplifying the gating strategy is provided in the Supplementary Information.
